# *Enterobacter* *hormaechei* subsp. *hoffmannii* subsp. nov., *Enterobacter hormaechei *subsp. *xiangfangensis* comb. nov., *Enterobacter roggenkampii* sp. nov., and *Enterobacter muelleri* is a later heterotypic synonym of *Enterobacter asburiae* based on computational analysis of sequenced *Enterobacter* genomes.

**DOI:** 10.12688/f1000research.14566.2

**Published:** 2018-06-29

**Authors:** Granger G. Sutton, Lauren M. Brinkac, Thomas H. Clarke, Derrick E. Fouts

**Affiliations:** 1 J Craig Venter Institute, Rockville, MD, 20850, USA

**Keywords:** Enterobacter, hormaechei, steigerwaltii, oharae, xiangfangensis, hoffmannii, roggenkampii, Prokaryote Code

## Abstract

**Background:** The predominant species in clinical *Enterobacter* isolates is *E. hormaechei*. Many articles, clinicians, and GenBank submissions misname these strains as *E. cloacae*. The lack of sequenced type strains or named species/subspecies for some clades in the *E. cloacae* complex complicate the issue.

**Methods:** The genomes of the type strains for *Enterobacter hormaechei* subsp. *oharae*, *E*. *hormaechei *subsp. *steigerwaltii*, and *E. xiangfangensis*, and two strains from Hoffmann clusters III and IV of the *E. cloacae* complex were sequenced. These genomes, the *E*. *hormaechei* subsp. *hormaechei* type strain, and other available *Enterobacter* type strains were analysed in conjunction with all extant *Enterobacter* genomes in NCBI’s RefSeq using Average Nucleotide Identity (ANI).

**Results:** There were five recognizable subspecies of *E. hormaechei*: *E. hormaechei* subsp. *hoffmannii* subsp. nov., *E. hormaechei *subsp. *xiangfangensis* comb. nov., and the three previously known subspecies. One of the strains sequenced from the *E. cloacae* complex was not a novel *E. hormaechei* subspecies but rather a member of a clade of a novel species: *E. roggenkampii* sp. nov.. *E. muelleri* was determined to be a later heterotypic synonym of *E. asburiae* which should take precedence.

**Conclusion:** The phylogeny of the *Enterobacter* genus, particularly the *cloacae* complex, was re-evaluated based on the type strain genome sequences and all other available *Enterobacter* genomes in RefSeq.

## Introduction

The name *Enterobacter hormaechei* was created for a taxon at the rank of species that had previously been called Enteric Group 75. O’Hara *et al.* ^1^ defined the type strain to be ATCC 49162 ^T^ from the 23 strains they studied. Twelve of the strains were shown to be closely related via DNA-DNA hybridization (DDH) and less closely related to other *Enterobacter* species. Numerous biochemical assays were performed on the 23 strains to characterize and differentiate the new species.

Hoffmann and Roggenkamp ^2^ investigated the genetic structure of the *E. cloacae* complex (the set of species included in this complex has varied over time) by a combination of sequencing of the three housekeeping genes *hsp60*, *rpoB*, and *hemB;* and PCR-restriction fragment length polymorphism (PCR-RFLP) analysis of *ampC*. They defined 12 genetic clusters (I-XII) based most exhaustively on the *hsp60* sequencing. Three of the clusters (cluster III, 58 strains; cluster VI, 28 strains; and cluster VIII, 59 strains) accounted for 70% of the 206 strains studied. The authors noted that “Only 3% of our study strains clustered with the type strain of *E. cloacae*.” (cluster XI), “We found that 3% of our study strains clustered around the *E. hormaechei* type strain.” (cluster VII), and “Our clusters VI and VIII were closely related to *E. hormaechei* cluster VII. DDH studies are needed to verify whether these clusters form a common DNA relatedness group allowing emending and broadening of the species description of *E. hormaechei*.”.

Hoffmann *et al.* ^3^ followed up with a characterization of clusters VI, VII, and VIII asserting based on DDH that these clusters were subspecies of the same species. Since cluster VII contained the type strain for *E. hormaechei* Hoffmann *et al*. named cluster VII *E. hormaechei* subsp. *hormaechei*, cluster VI *E. hormaechei* subsp. *oharae*, and cluster VIII *E. hormaechei* subsp. *steigerwaltii*. Forty-eight strains were characterized using 129 biochemical tests showing that there were phenotypic differences between the subspecies. Unfortunately the authors did not decide to include the other predominant cluster (III) in their analysis, nor did they validly publish these subspecies names. This was rectified recently in Validation List no. 172 ^4^.

Gu *et al.* ^5^ defined *E. xiangfangensis* using a phylogenetic tree based upon concatenated partial *rpoB*, *atpD*, *gyrB* and *infB* gene sequences from a novel isolate and existing type strains where *E. xiangfangensis* grouped closest to *E. hormaechei*. Biochemical assays were performed and *E. xiangfangensis* strains were differentiable from the *E. hormaechei* type strain.

During analysis of the *E. cloacae* complex and *E.*(now *Klebsiella* ^6^) *aerogenes* strains looking at antimicrobial resistance patterns ^7^, many of the Hoffmann *et al*. clusters were rediscovered using whole genome comparisons such as SNP analysis and average nucleotide identity (ANI). The clusters were identifiable by the *hsp60* sequences deposited by the Hoffmann group. The three subspecies of *E. hormaechei* defined by Hoffmann *et al*. fell within the expected ANI range for bacterial species, being greater than 95% ANI between subspecies and greater than 98% ANI within a subspecies. Unexpectedly Hoffmann cluster III also met the ANI criteria to be an *E. hormaechei* subspecies. Further, genomes named *E. xiangfangensis* in GenBank fell within the *E. hormaechei* subsp. *steigerwaltii* cluster rather than a separate cluster. Moreover, most of the genomes in these clusters were mistakenly identified as *E. cloacae* when they were submitted to GenBank. To resolve the naming inconsistencies of these genomes the type strains for *E. hormaechei* subsp. *steigerwaltii*, *E. hormaechei* subsp. *oharae*, *E. xiangfangensis*, Hoffmann cluster III, and Hoffmann cluster IV were sequenced.

Tools for bacterial species assignment have changed over time ^8, 9^. Initially, morphology as viewed through a microscope and later aided by staining such as Gram staining ^10^ to distinguish cell wall differences was used. Biochemical assays and other methods to determine phenotype followed. Use of the genome started with DNA-DNA hybridization (DDH) where a 70% threshold for species followed later by a 79% threshold for subspecies were proposed. Widespread use of marker genes in particular the 16S rRNA gene made assays easier. A threshold of less than 97% identity for the 16S rRNA gene was used to determine a new species but values above 97% could not guarantee that isolates were the same species. The sequence of other less conserved marker genes such as hsp60 has also been used to differentiate species. More recently multiple marker genes are sequenced and a combined alignment is used. With the advent of inexpensive genome sequencing, computing ANI, which correlates very closely with DDH, has largely supplanted other methods. Studies have shown that an ANI threshold between 94-96.5% correlates well with existing species definitions and 97-98% for subspecies ^11– 19^. DDH has been shown to not only correlate with ANI but also with how many of the genes or what fraction of the genomes are shared in common so some ANI based tools take this measurement into account as well ^17– 19^. Most definitions of new species involve sequencing the genome and taking ANI and shared gene content into account in some fashion but many species definitions predate genome sequencing and some type strains have not been sequenced. There is no generally accepted method for reconciling older species definitions with genome comparisons but usually ANI and shared gene content form a basis for the analysis.

As Hoffmann ^2, 3^ and others ^20– 26^ discovered the predominant species in clinical *Enterobacter* isolates is *E. hormaechei*. Unfortunately many articles, clinicians, and GenBank submissions misname these strains as *E. cloacae* perhaps as a short hand for the *E. cloacae* complex and possibly due to the *E. hormaechei* subspecies not being validly published until recently. Another issue was the lack of sequenced type strains or named species/subspecies for some clades. The definition of what species/subspecies make up the *E. cloacae* complex has been in flux ^2, 27, 28^ and even what species are in the genus *Enterobacter* ^29– 31^.

The *E. cloacae* complex was shown to have 18 clades (A-R) ^7^, 12 of which corresponded to 11 of the 12 clusters defined previously by Hoffmann ^2^. Hoffmann cluster X is *E. nimipressuralis* which has been reclassified as *Lelliottia nimipressuralis* ^29^. Table 1 incorporates more recently sequenced genomes and published papers adding four clades (S-V) and incorporating the latest literature. For example, clade R (Hoffmann cluster IX) was recently defined to be *E. bugandensis* ^31^.

**Table 1.** Type and proxy strain genomes for *Enterobacter cloacae* complex clades. *E. lignolyticus* and *E. timonensis* have not been validly published and are deemed to be outside of the *E. cloacae* complex. *E. siamensis* and *E. tabaci* do not have sequenced genomes but based on their 16S rRNA genes may be in the *E. cloacae* complex. Proxy indicates whether a type or proxy strain was available. The last two columns are for the clade (A-V) and Hoffmann cluster (I-XII).

Short ID	BioSample ID	Current name	Proposed name	Strain	Proxy		
ATCC35953	SAMN03742638	*E. asburiae*	*E. asburiae*	ATCC 35953	type	J	I
obactermuelleri	SAMEA103972944	*E. muelleri*	*E. asburiae*	JM-458	type	J	I
cterbugandensis	SAMEA104115216	*E. bugandensis*	*E. bugandensis*	EB-247	type	R	IX
tercancerogenus	SAMEA104113916	*E. cancerogenus*	*E. cancerogenus*	ATCC 33241	type	U	
1161ECLO	SAMN03197118	*E. cloacae*	*E. cloacae* complex clade K	1161_ECLO	proxy	K	
GN02587	SAMN03732717	*E. cloacae* complex sp. GN02587	*E. cloacae* complex clade L	GN02587	proxy	L	
DS11005	SAMN07448201	*E. cloacae*	*E. cloacae* complex clade N	DS11005	proxy	N	
GN05526	SAMN04578342	*E. cloacae* complex sp. GN05526	*E. cloacae* complex clade O	GN05526	proxy	O	
624ECLO	SAMN03197824	*E. cloacae*	*E. cloacae* complex clade P	624_ECLO	proxy	P	
ND22	SAMN05212257	*E. cloacae*	*E. cloacae* complex clade S	ND22	proxy	S	
C9	SAMN06237083	*E. cancerogenus*	*E. cloacae* complex clade T	C9	proxy	T	
ATCC13047	SAMN02603901	*E. cloacae* ssp. *cloacae*	*E. cloacae* ssp. *cloacae*	ATCC 13047	type	G	XI
SDM	SAMN02603521	*E. cloacae* ssp. *dissolvens*	*E. cloacae* ssp. *dissolven*s	SDM	proxy	H	XII
DSM14563	SAMN05581748	*E. cloacae* complex Hoffmann cluster III	*E. hormaechei* ssp. *hoffmannii*	DSM 14563	type	D	III
ATCC49162	SAMN05787340	*E. hormaechei* ssp. *hormaechei*	*E. hormaechei* ssp. *hormaechei*	ATCC 49162	type	E	VII
DSM16687	SAMN05581749	*E. hormaechei* ssp. *oharae*	*E. hormaechei* ssp. *oharae*	DSM 16687	type	C	VI
DSM16691	SAMN05581751	*E. hormaechei* ssp. *steigerwaltii*	*E. hormaechei* ssp. *steigerwaltii*	DSM 16691	type	B	VIII
LMG27195	SAMN05581746	*E. xiangfangensis*	*E. hormaechei* ssp. *xiangfangensis*	LMG27195	type	A	VI
DSM13645	SAMN05581747	*E. kobei*	*E. kobei*	DSM 13645	type	Q	II
EN119	SAMN05787341	*E. ludwigii*	*E. ludwigii*	EN-119	type	I	V
LMG25706	SAMN02471025	*E. mori*	*E. mori*	LMG 25706	type	F	
DSM16690	SAMN05581750	*E. cloacae* complex Hoffmann cluster IV	*E. roggenkampii*	DSM 16690	type	M	IV
nterobactersoli	SAMEA104113920	*E. soli*	*E. soli*	LMG 25861	type	V	
SCF1	SAMN00116754	*E. lignolyticus*	*E. lignolyticus*	SCF1	type		
mt20	SAMEA3859023	*E. timonensis*	*E. timonensis*	mt20	type		
	No genome	*E. siamensis*					
	No genome	*E. tabaci*					

## Results

**Figure 1.**
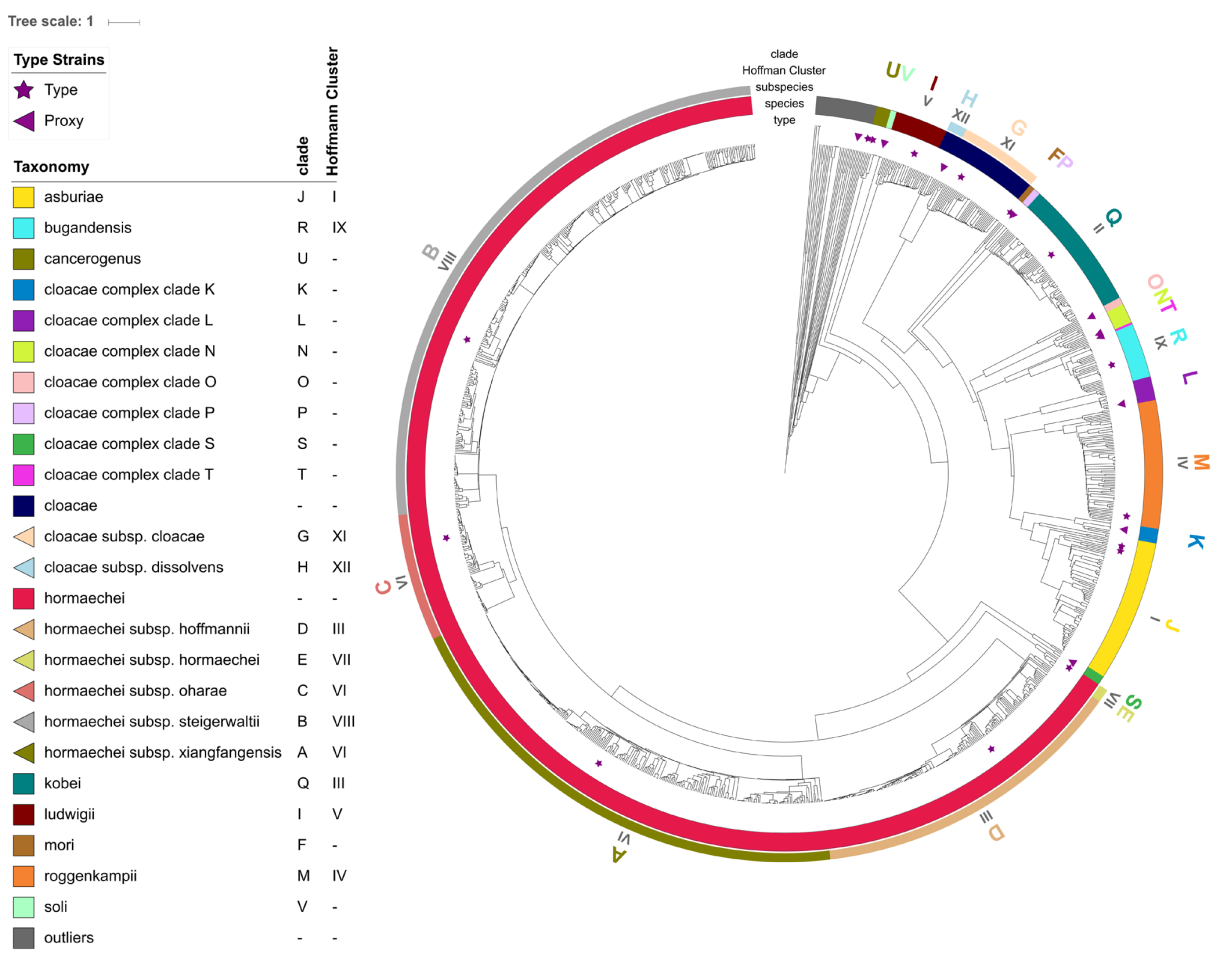
Average nucleotide identity (ANI) based tree for 1,249 NCBI RefSeq *Enterobacter* labelled genomes.

All RefSeq genomes labelled as being in the genus *Enterobacter* were downloaded from NCBI RefSeq resulting in 1,249 genomes. A fast approximate ANI tool, called MASH ^32^, was used to generate a pairwise ANI based distance matrix and average linkage hierarchical clustering was used to generate the tree shown in Figure 1. 1,216 genomes were assigned to 22 clades (A-V Table 1) in the *E. cloacae* complex ( Supplemental Table 1) while 30 genomes were deemed to be outliers and not in the *Enterobacter* genus (best MASH matches in Supplemental Table 2) as well as 2 *E. lignolyticus* genomes and 1 *E. timonensis* genome deemed to be outside of the *E. cloacae* complex. Two species of *Enterobacter*: *E. siamensis* and *E. tabaci* do not have sequenced genomes and their type strains’ 16S rRNA sequences while having full length matches at 98% and 99% respectively to some *E. cloacae* complex genomes did not have definitive matches to any particular clade. The type strains for *E. asburiae* and *E. muelleri* fall within the same clade (J – Hoffmann cluster I). All 78 genomes in this clade are above the 95% ANI species cut-off ( Table 2) but using a 98% ANI subspecies cut-off produces 8 subclades of sizes 1, 1, 2, 2, 2 ( *E. muelleri*), 3 ( *E. asburiae*), 24, and 43. Thus *E. muelleri* ^33^ is a later heterotypic synonym of *E. asburiae* ^34^ which should take precedence. Whether the 8 subclades of *E. asburiae* should be treated as subspecies is beyond the scope of this paper but is revisited in the Discussion section.

**Table 2.** Pairwise Average nucleotide identity (ANI) values within and between the *Enterobacter cloacae* complex clades. Mean and standard deviation are shown above and the minimum and maximum pairwise values below. The last two rows show *E. lignolyticus* (Li) and *E. timonensis* (Ti) which have consistently lower ANI values.

	A	B	C	D	E	F	G	H	I	J	K	L	M	N	O	P	Q	R	S	T	U	V
A	98.77 ±0.46 (97.9- 100)	96.96 ±0.13 (96.2- 97.5)	97.01 ±0.13 (96.3- 97.6)	96.17 ±0.15 (95.3- 96.9)	94.53 ±0.18 (93.9- 95.2)	89.80 ±0.32 (88.9- 91.0)	88.63 ±0.43 (87.5- 90.9)	88.18 ±0.29 (87.1- 89.4)	87.65 ±0.31 (86.3- 88.8)	89.49 ±0.40 (87.8- 91.2)	89.39 ±0.28 (88.4- 90.3)	89.16 ±0.28 (88.0- 90.3)	89.87 ±0.37 (88.4- 91.6)	89.15 ±0.29 (88.1- 90.4)	89.11 ±0.40 (88.3- 90.7)	87.86 ±0.36 (86.8- 88.7)	89.64 ±0.35 (88.5- 91.2)	90.03 ±0.29 (89.0- 91.2)	93.77 ±0.19 (93.2- 94.5)	89.85 ±0.15 (89.3- 90.4)	86.89 ±0.38 (85.3- 88.1)	87.43 ±0.23 (86.9- 88.2)
B	96.96 ±0.13 (96.2- 97.5)	98.61 ±0.29 (97.8- 100)	97.33 ±0.13 (96.7- 97.8)	95.98 ±0.17 (95.3- 96.9)	94.51 ±0.21 (94.0- 95.2)	89.48 ±0.41 (88.5- 90.8)	88.48 ±0.42 (87.0- 90.6)	88.14 ±0.37 (86.8- 89.4)	88.28 ±0.33 (87.0- 89.5)	89.28 ±0.44 (87.5- 91.1)	88.98 ±0.27 (88.1- 89.9)	89.13 ±0.30 (88.1- 90.1)	89.43 ±0.44 (87.9- 91.3)	89.13 ±0.28 (88.3- 90.2)	89.09 ±0.39 (88.2- 90.5)	87.92 ±0.55 (86.6- 89.1)	89.43 ±0.36 (88.3- 91.1)	90.01 ±0.29 (89.1- 91.3)	93.89 ±0.25 (93.0- 94.6)	89.57 ±0.24 (89.0- 90.2)	87.27 ±0.42 (85.9- 88.3)	87.65 ±0.25 (86.9- 88.4)
C	97.01 ±0.13 (96.3- 97.6)	97.33 ±0.13 (96.7- 97.8)	98.66 ±0.84 (97.6- 100)	96.03 ±0.16 (95.5- 96.6)	94.75 ±0.16 (94.4- 95.2)	89.35 ±0.39 (88.5- 90.5)	88.84 ±0.43 (87.9- 90.6)	88.25 ±0.32 (87.3- 88.9)	88.10 ±0.30 (87.3- 89.1)	89.29 ±0.44 (87.9- 90.8)	89.21 ±0.31 (88.1- 89.8)	89.19 ±0.26 (88.4- 89.9)	89.54 ±0.47 (88.0- 91.4)	89.33 ±0.24 (88.5- 90.0)	89.08 ±0.36 (88.1- 90.3)	88.23 ±0.49 (87.3- 89.0)	89.53 ±0.39 (88.5- 91.2)	89.93 ±0.25 (89.0- 91.1)	93.98 ±0.25 (93.4- 94.6)	89.95 ±0.27 (89.4- 90.3)	87.50 ±0.48 (86.3- 88.7)	87.80 ±0.32 (87.1- 88.3)
D	96.17 ±0.15 (95.3- 96.9)	95.98 ±0.17 (95.3- 96.9)	96.03 ±0.16 (95.5- 96.6)	98.89 ±0.51 (97.7- 100)	94.18 ±0.16 (93.7- 94.7)	89.54 ±0.35 (88.8- 90.8)	88.79 ±0.42 (88.0- 90.6)	88.47 ±0.31 (87.8- 89.4)	87.71 ±0.27 (86.7- 89.1)	89.53 ±0.39 (88.2- 91.2)	88.94 ±0.42 (87.8- 89.8)	89.11 ±0.20 (88.5- 90.0)	89.69 ±0.41 (88.4- 91.5)	89.14 ±0.31 (88.3- 90.3)	88.96 ±0.38 (88.3- 90.6)	88.30 ±0.44 (87.2- 89.1)	89.08 ±0.39 (88.1- 91.0)	90.19 ±0.29 (89.1- 91.7)	93.96 ±0.14 (93.5- 94.5)	89.86 ±0.18 (89.4- 90.4)	87.14 ±0.39 (85.9- 88.4)	87.75 ±0.22 (87.3- 88.4)
E	94.53 ±0.18 (93.9- 95.2)	94.51 ±0.21 (94.0- 95.2)	94.75 ±0.16 (94.4- 95.2)	94.18 ±0.16 (93.7- 94.7)	99.08 ±0.54 (98.2- 100)	88.89 ±0.34 (88.4- 89.7)	88.51 ±0.34 (87.8- 89.9)	88.01 ±0.32 (87.4- 88.9)	87.30 ±0.40 (86.5- 88.4)	88.85 ±0.39 (87.5- 91.0)	88.40 ±0.49 (87.5- 89.6)	88.45 ±0.24 (87.8- 88.9)	89.03 ±0.44 (87.8- 90.5)	88.55 ±0.44 (87.6- 89.6)	88.48 ±0.45 (87.8- 89.5)	87.41 ±0.46 (86.7- 88.4)	89.28 ±0.25 (88.5- 90.0)	89.98 ±0.30 (89.4- 90.8)	93.32 ±0.24 (92.8- 93.9)	89.95 ±0.21 (89.7- 90.3)	87.22 ±0.53 (85.9- 88.3)	87.19 ±0.14 (86.9- 87.5)
F	89.80 ±0.32 (88.9- 91.0)	89.48 ±0.41 (88.5- 90.8)	89.35 ±0.39 (88.5- 90.5)	89.54 ±0.35 (88.8- 90.8)	88.89 ±0.34 (88.4- 89.7)	97.79 ±0.44 (97.4- 98.3)	89.10 ±0.44 (88.1- 90.7)	89.60 ±0.27 (89.2- 90.1)	88.82 ±0.22 (88.4- 89.3)	91.22 ±0.31 (90.3- 92.1)	91.08 ±0.29 (90.6- 91.6)	90.20 ±0.24 (89.8- 90.6)	90.85 ±0.33 (90.1- 91.5)	90.20 ±0.35 (89.6- 90.9)	91.33 ±0.42 (90.4- 91.8)	89.49 ±0.18 (89.2- 89.7)	90.60 ±0.28 (90.0- 91.3)	91.40 ±0.21 (90.8- 92.0)	89.23 ±0.22 (88.8- 89.6)	91.19 ±0.21 (91.0- 91.4)	88.69 ±0.34 (88.0- 89.2)	87.98 ±0.35 (87.5- 88.3)
G	88.63 ±0.43 (87.5- 90.9)	88.48 ±0.42 (87.0- 90.6)	88.84 ±0.43 (87.9- 90.6)	88.79 ±0.42 (88.0- 90.6)	88.51 ±0.34 (87.8- 89.9)	89.10 ±0.44 (88.1- 90.7)	98.42 ±0.31 (97.7- 100)	95.70 ±0.18 (95.2- 96.2)	88.82 ±0.31 (87.6- 89.5)	89.93 ±0.38 (88.8- 91.3)	89.18 ±0.32 (88.5- 90.2)	89.47 ±0.25 (89.0- 90.4)	89.28 ±0.39 (88.0- 90.9)	90.14 ±0.31 (89.3- 91.1)	90.00 ±0.28 (89.3- 90.9)	88.65 ±0.29 (87.7- 89.3)	89.86 ±0.32 (88.7- 91.5)	89.75 ±0.34 (88.9- 91.2)	88.13 ±0.31 (87.5- 88.7)	89.99 ±0.17 (89.6- 90.4)	87.86 ±0.20 (87.3- 88.3)	87.48 ±0.19 (87.0- 88.0)
H	88.18 ±0.29 (87.1- 89.4)	88.14 ±0.37 (86.8- 89.4)	88.25 ±0.32 (87.3- 88.9)	88.47 ±0.31 (87.8- 89.4)	88.01 ±0.32 (87.4- 88.9)	89.60 ±0.27 (89.2- 90.1)	95.70 ±0.18 (95.2- 96.2)	98.82 ±0.24 (98.6- 100)	89.09 ±0.30 (88.2- 89.8)	89.97 ±0.38 (88.7- 90.9)	89.85 ±0.37 (89.0- 90.5)	89.69 ±0.25 (89.2- 90.1)	89.47 ±0.45 (88.2- 90.6)	90.43 ±0.33 (89.5- 91.0)	90.78 ±0.19 (90.4- 91.1)	88.75 ±0.30 (88.2- 89.4)	89.35 ±0.34 (88.3- 90.1)	90.13 ±0.33 (89.3- 91.1)	88.25 ±0.22 (87.7- 88.7)	90.61 ±0.16 (90.3- 90.8)	88.24 ±0.31 (87.6- 89.0)	88.15 ±0.19 (87.8- 88.4)
I	87.65 ±0.31 (86.3- 88.8)	88.28 ±0.33 (87.0- 89.5)	88.10 ±0.30 (87.3- 89.1)	87.71 ±0.27 (86.7- 89.1)	87.30 ±0.40 (86.5- 88.4)	88.82 ±0.22 (88.4- 89.3)	88.82 ±0.31 (87.6- 89.5)	89.09 ±0.30 (88.2- 89.8)	98.63 ±0.24 (98.0- 100)	88.98 ±0.33 (87.7- 89.9)	89.01 ±0.24 (88.3- 89.6)	88.65 ±0.33 (87.8- 89.7)	88.90 ±0.35 (87.8- 89.9)	88.87 ±0.33 (88.0- 89.6)	89.17 ±0.27 (88.5- 89.8)	88.46 ±0.38 (87.7- 89.1)	89.32 ±0.27 (88.5- 90.1)	88.95 ±0.33 (87.9- 89.6)	87.44 ±0.40 (86.5- 88.6)	88.90 ±0.20 (88.3- 89.3)	87.17 ±0.23 (86.5- 87.7)	87.64 ±0.18 (87.3- 88.0)
J	89.49 ±0.40 (87.8- 91.2)	89.28 ±0.44 (87.5- 91.1)	89.29 ±0.44 (87.9- 90.8)	89.53 ±0.39 (88.2- 91.2)	88.85 ±0.39 (87.5- 91.0)	91.22 ±0.31 (90.3- 92.1)	89.93 ±0.38 (88.8- 91.3)	89.97 ±0.38 (88.7- 90.9)	88.98 ±0.33 (87.7- 89.9)	97.20 ± 1.15 (94.5- 100)	94.38 ±0.28 (93.6- 95.2)	93.50 ±0.21 (92.8- 94.0)	93.47 ±0.25 (92.5- 94.3)	91.88 ±0.29 (91.1- 92.6)	92.99 ±0.29 (92.2- 94.0)	91.93 ±0.27 (91.3- 92.7)	92.03 ±0.26 (91.2- 93.0)	92.30 ±0.33 (91.1- 93.3)	89.13 ±0.41 (87.5- 90.0)	92.46 ±0.25 (91.8- 92.9)	87.79 ±0.46 (86.0- 88.8)	88.17 ±0.26 (87.5- 88.7)
K	89.39 ±0.28 (88.4- 90.3)	88.98 ±0.27 (88.1- 89.9)	89.21 ±0.31 (88.1- 89.8)	88.94 ±0.42 (87.8- 89.8)	88.40 ±0.49 (87.5- 89.6)	91.08 ±0.29 (90.6- 91.6)	89.18 ±0.32 (88.5- 90.2)	89.85 ±0.37 (89.0- 90.5)	89.01 ±0.24 (88.3- 89.6)	94.38 ±0.28 (93.6- 95.2)	98.26 ±0.77 (97.5- 100)	93.29 ±0.26 (92.8- 93.7)	94.14 ±0.36 (93.3- 95.5)	92.05 ±0.44 (90.9- 92.6)	92.90 ±0.24 (92.5- 93.3)	91.78 ±0.21 (91.4- 92.1)	91.63 ±0.28 (90.5- 92.2)	92.38 ±0.27 (91.6- 92.9)	89.18 ±0.30 (88.7- 89.8)	92.37 ±0.22 (92.0- 92.7)	87.62 ±0.34 (86.8- 88.2)	87.65 ±0.16 (87.3- 87.8)
L	89.16 ±0.28 (88.0- 90.3)	89.13 ±0.30 (88.1- 90.1)	89.19 ±0.26 (88.4- 89.9)	89.11 ±0.20 (88.5- 90.0)	88.45 ±0.24 (87.8- 88.9)	90.20 ±0.24 (89.8- 90.6)	89.47 ±0.25 (89.0- 90.4)	89.69 ±0.25 (89.2- 90.1)	88.65 ±0.33 (87.8- 89.7)	93.50 ±0.21 (92.8- 94.0)	93.29 ±0.26 (92.8- 93.7)	97.80 ±1.94 (95.6- 100)	93.20 ±0.21 (92.5- 93.8)	91.67 ±0.27 (91.0- 92.2)	92.24 ±0.13 (92.0- 92.5)	90.87 ±0.21 (90.5- 91.3)	91.93 ±0.23 (91.2- 92.7)	91.65 ±0.25 (91.0- 92.4)	89.15 ±0.26 (88.6- 89.7)	91.26 ±0.25 (90.8- 91.6)	88.06 ±0.32 (87.2- 88.7)	87.73 ±0.28 (87.0- 88.1)
M	89.87 ±0.37 (88.4- 91.6)	89.43 ±0.44 (87.9- 91.3)	89.54 ±0.47 (88.0- 91.4)	89.69 ±0.41 (88.4- 91.5)	89.03 ±0.44 (87.8- 90.5)	90.85 ±0.33 (90.1- 91.5)	89.28 ±0.39 (88.0- 90.9)	89.47 ±0.45 (88.2- 90.6)	88.90 ±0.35 (87.8- 89.9)	93.47 ±0.25 (92.5- 94.3)	94.14 ±0.36 (93.3- 95.5)	93.20 ±0.21 (92.5- 93.8)	97.72 ±0.86 (95.1- 100)	92.30 ±0.30 (91.5- 93.1)	92.39 ±0.28 (91.6- 93.0)	90.92 ±0.27 (90.1- 91.4)	91.47 ±0.34 (90.2- 92.9)	92.21 ±0.29 (91.1- 93.0)	89.57 ±0.36 (88.5- 90.5)	91.90 ±0.22 (91.3- 92.3)	87.31 ±0.44 (86.2- 88.3)	87.72 ±0.26 (87.3- 88.3)
N	89.15 ±0.29 (88.1- 90.4)	89.13 ±0.28 (88.3- 90.2)	89.33 ±0.24 (88.5- 90.0)	89.14 ±0.31 (88.3- 90.3)	88.55 ±0.44 (87.6- 89.6)	90.20 ±0.35 (89.6- 90.9)	90.14 ±0.31 (89.3- 91.1)	90.43 ±0.33 (89.5- 91.0)	88.87 ±0.33 (88.0- 89.6)	91.88 ±0.29 (91.1- 92.6)	92.05 ±0.44 (90.9- 92.6)	91.67 ±0.27 (91.0- 92.2)	92.30 ±0.30 (91.5- 93.1)	98.28 ±0.41 (97.6- 99.9)	93.13 ±0.20 (92.8- 93.5)	90.64 ±0.16 (90.2- 90.9)	90.78 ±0.30 (90.0- 91.5)	91.14 ±0.28 (90.3- 91.8)	88.72 ±0.41 (87.7- 89.5)	90.90 ±0.39 (90.3- 91.5)	86.75 ±0.38 (85.7- 87.4)	87.37 ±0.14 (87.2- 87.7)
O	89.11 ±0.40 (88.3- 90.7)	89.09 ±0.39 (88.2- 90.5)	89.08 ±0.36 (88.1- 90.3)	88.96 ±0.38 (88.3- 90.6)	88.48 ±0.45 (87.8- 89.5)	91.33 ±0.42 (90.4- 91.8)	90.00 ±0.28 (89.3- 90.9)	90.78 ±0.19 (90.4- 91.1)	89.17 ±0.27 (88.5- 89.8)	92.99 ±0.29 (92.2- 94.0)	92.90 ±0.24 (92.5- 93.3)	92.24 ±0.13 (92.0- 92.5)	92.39 ±0.28 (91.6- 93.0)	93.13 ±0.20 (92.8- 93.5)	97.90 ±0.88 (97.0- 98.8)	91.05 ±0.27 (90.5- 91.4)	91.77 ±0.28 (91.0- 92.5)	91.74 ±0.20 (91.3- 92.2)	89.07 ±0.36 (88.5- 89.8)	91.42 ±0.21 (91.2- 91.6)	88.02 ±0.36 (87.3- 88.7)	87.80 ±0.14 (87.6- 88.0)
P	87.86 ±0.36 (86.8- 88.7)	87.92 ±0.55 (86.6- 89.1)	88.23 ±0.49 (87.3- 89.0)	88.30 ±0.44 (87.2- 89.1)	87.41 ±0.46 (86.7- 88.4)	89.49 ±0.18 (89.2- 89.7)	88.65 ±0.29 (87.7- 89.3)	88.75 ±0.30 (88.2- 89.4)	88.46 ±0.38 (87.7- 89.1)	91.93 ±0.27 (91.3- 92.7)	91.78 ±0.21 (91.4- 92.1)	90.87 ±0.21 (90.5- 91.3)	90.92 ±0.27 (90.1- 91.4)	90.64 ±0.16 (90.2- 90.9)	91.05 ±0.27 (90.5- 91.4)	98.77 ±0.91 (98.2- 100)	90.44 ±0.36 (89.7- 91.2)	90.34 ±0.37 (89.6- 91.1)	88.14 ±0.40 (87.5- 88.9)	90.00 ±0.26 (89.8- 90.2)	86.49 ±0.45 (85.7- 87.2)	88.32 ±0.19 (88.1- 88.5)
Q	89.64 ±0.35 (88.5- 91.2)	89.43 ±0.36 (88.3- 91.1)	89.53 ±0.39 (88.5- 91.2)	89.08 ±0.39 (88.1- 91.0)	89.28 ±0.25 (88.5- 90.0)	90.60 ±0.28 (90.0- 91.3)	89.86 ±0.32 (88.7- 91.5)	89.35 ±0.34 (88.3- 90.1)	89.32 ±0.27 (88.5- 90.1)	92.03 ±0.26 (91.2- 93.0)	91.63 ±0.28 (90.5- 92.2)	91.93 ±0.23 (91.2- 92.7)	91.47 ±0.34 (90.2- 92.9)	90.78 ±0.30 (90.0- 91.5)	91.77 ±0.28 (91.0- 92.5)	90.44 ±0.36 (89.7- 91.2)	98.67 ±0.39 (97.9- 100)	92.01 ±0.25 (91.2- 92.9)	89.12 ±0.30 (88.4- 89.9)	91.89 ±0.20 (91.3- 92.3)	88.42 ±0.34 (87.2- 89.0)	87.40 ±0.21 (87.0- 88.0)
R	90.03 ±0.29 (89.0- 91.2)	90.01 ±0.29 (89.1- 91.3)	89.93 ±0.25 (89.0- 91.1)	90.19 ±0.29 (89.1- 91.7)	89.98 ±0.30 (89.4- 90.8)	91.40 ±0.21 (90.8- 92.0)	89.75 ±0.34 (88.9- 91.2)	90.13 ±0.33 (89.3- 91.1)	88.95 ±0.33 (87.9- 89.6)	92.30 ±0.33 (91.1- 93.3)	92.38 ±0.27 (91.6- 92.9)	91.65 ±0.25 (91.0- 92.4)	92.21 ±0.29 (91.1- 93.0)	91.14 ±0.28 (90.3- 91.8)	91.74 ±0.20 (91.3- 92.2)	90.34 ±0.37 (89.6- 91.1)	92.01 ±0.25 (91.2- 92.9)	98.24 ±0.79 (95.6- 100)	90.92 ±0.24 (90.2- 91.4)	94.18 ±0.12 (94.0- 94.6)	88.49 ±0.34 (87.5- 89.3)	88.37 ±0.21 (87.9- 88.7)
S	93.77 ±0.19 (93.2- 94.5)	93.89 ±0.25 (93.0- 94.6)	93.98 ±0.25 (93.4- 94.6)	93.96 ±0.14 (93.5- 94.5)	93.32 ±0.24 (92.8- 93.9)	89.23 ±0.22 (88.8- 89.6)	88.13 ±0.31 (87.5- 88.7)	88.25 ±0.22 (87.7- 88.7)	87.44 ±0.40 (86.5- 88.6)	89.13 ±0.41 (87.5- 90.0)	89.18 ±0.30 (88.7- 89.8)	89.15 ±0.26 (88.6- 89.7)	89.57 ±0.36 (88.5- 90.5)	88.72 ±0.41 (87.7- 89.5)	89.07 ±0.36 (88.5- 89.8)	88.14 ±0.40 (87.5- 88.9)	89.12 ±0.30 (88.4- 89.9)	90.92 ±0.24 (90.2- 91.4)	98.52 ±0.89 (97.8- 100)	89.75 ±0.29 (89.5- 90.2)	88.18 ±0.32 (87.4- 88.7)	87.66 ±0.33 (87.2- 88.1)
T	89.85 ±0.15 (89.3- 90.4)	89.57 ±0.24 (89.0- 90.2)	89.95 ±0.27 (89.4- 90.3)	89.86 ±0.18 (89.4- 90.4)	89.95 ±0.21 (89.7- 90.3)	91.19 ±0.21 (91.0- 91.4)	89.99 ±0.17 (89.6- 90.4)	90.61 ±0.16 (90.3- 90.8)	88.90 ±0.20 (88.3- 89.3)	92.46 ±0.25 (91.8- 92.9)	92.37 ±0.22 (92.0- 92.7)	91.26 ±0.25 (90.8- 91.6)	91.90 ±0.22 (91.3- 92.3)	90.90 ±0.39 (90.3- 91.5)	91.42 ±0.21 (91.2- 91.6)	90.00 ±0.26 (89.8- 90.2)	91.89 ±0.20 (91.3- 92.3)	94.18 ±0.12 (94.0- 94.6)	89.75 ±0.29 (89.5- 90.2)	100 ±0.00 (100- 100)	88.62 ±0.40 (87.7- 88.9)	88.76 ±0.04 (88.7- 88.8)
U	86.89 ±0.38 (85.3- 88.1)	87.27 ±0.42 (85.9- 88.3)	87.50 ±0.48 (86.3- 88.7)	87.14 ±0.39 (85.9- 88.4)	87.22 ±0.53 (85.9- 88.3)	88.69 ±0.34 (88.0- 89.2)	87.86 ±0.20 (87.3- 88.3)	88.24 ±0.31 (87.6- 89.0)	87.17 ±0.23 (86.5- 87.7)	87.79 ±0.46 (86.0- 88.8)	87.62 ±0.34 (86.8- 88.2)	88.06 ±0.32 (87.2- 88.7)	87.31 ±0.44 (86.2- 88.3)	86.75 ±0.38 (85.7- 87.4)	88.02 ±0.36 (87.3- 88.7)	86.49 ±0.45 (85.7- 87.2)	88.42 ±0.34 (87.2- 89.0)	88.49 ±0.34 (87.5- 89.3)	88.18 ±0.32 (87.4- 88.7)	88.62 ±0.40 (87.7- 88.9)	98.80 ±0.65 (98.3- 100)	86.62 ±0.27 (86.2- 86.9)
V	87.43 ±0.23 (86.9- 88.2)	87.65 ±0.25 (86.9- 88.4)	87.80 ±0.32 (87.1- 88.3)	87.75 ±0.22 (87.3- 88.4)	87.19 ±0.14 (86.9- 87.5)	87.98 ±0.35 (87.5- 88.3)	87.48 ±0.19 (87.0- 88.0)	88.15 ±0.19 (87.8- 88.4)	87.64 ±0.18 (87.3- 88.0)	88.17 ±0.26 (87.5- 88.7)	87.65 ±0.16 (87.3- 87.8)	87.73 ±0.28 (87.0- 88.1)	87.72 ±0.26 (87.3- 88.3)	87.37 ±0.14 (87.2- 87.7)	87.80 ±0.14 (87.6- 88.0)	88.32 ±0.19 (88.1- 88.5)	87.40 ±0.21 (87.0- 88.0)	88.37 ±0.21 (87.9- 88.7)	87.66 ±0.33 (87.2- 88.1)	88.76 ±0.04 (88.7- 88.8)	86.62 ±0.27 (86.2- 86.9)	99.99 ±0.00 (100- 100)
Li	82.06 ±0.30 (81.1- 83.0)	82.87 ±0.33 (81.8- 83.8)	83.03 ±0.16 (82.6- 83.5)	82.22 ±0.25 (81.4- 83.0)	84.03 ±0.17 (83.8- 84.3)	82.55 ±0.30 (82.2- 82.8)	82.25 ±0.61 (80.9- 83.3)	81.56 ±0.33 (81.1- 82.0)	81.73 ±0.39 (80.9- 82.6)	82.36 ±0.34 (81.6- 83.5)	83.02 ±0.31 (82.6- 83.3)	82.41 ±0.15 (82.2- 82.6)	82.68 ±0.49 (81.4- 83.5)	81.49 ±0.45 (80.9- 82.4)	83.16 ±0.34 (83.0- 83.7)	81.12 ±0.28 (80.9- 81.4)	81.61 ±0.21 (81.1- 82.0)	82.76 ±0.31 (81.8- 83.3)	83.72 ±0.31 (83.3- 84.1)	82.99 ±0.00 (83.0- 83.0)	83.13 ±0.31 (82.4- 83.5)	82.23 ±0.00 (82.2- 82.2)
Ti	85.07 ±0.27 (83.8- 85.9)	85.82 ±0.25 (85.1- 86.4)	85.45 ±0.24 (85.1- 86.3)	85.93 ±0.18 (85.2- 86.6)	84.70 ±0.40 (84.0- 85.3)	85.22 ±0.18 (85.1- 85.4)	84.47 ±0.32 (83.8- 85.1)	83.99 ±0.40 (83.3- 84.7)	83.88 ±0.23 (83.3- 84.3)	85.45 ±0.44 (84.3- 86.3)	84.59 ±0.40 (84.1- 85.2)	85.73 ±0.52 (84.9- 86.2)	85.10 ±0.39 (84.0- 86.0)	84.93 ±0.33 (84.3- 85.3)	85.42 ±0.22 (85.2- 85.7)	83.50 ±0.19 (83.3- 83.7)	84.71 ±0.37 (84.0- 85.5)	84.97 ±0.22 (84.7- 85.5)	86.16 ±0.16 (86.0- 86.4)	84.94 ±0.00 (84.9- 84.9)	85.58 ±0.17 (85.3- 85.8)	83.87 ±0.09 (83.8- 84.0)

Five clades (A-E) are above the 95% ANI cut-off to be considered the same species ( Table 2). Almost all within-clade pairwise ANIs are greater than between-clade ANIs ( Table 2) and all genomes within a clade had the highest pairwise ANI to the type strain for that clade, supporting that these are distinct subspecies. Based on *hsp60* sequences, clade A containing the *E. xiangfangensis* type strain is Hoffmann cluster VI; clade B containing the *E. hormaechei* subsp. *steigerwaltii* type strain is Hoffmann cluster VIII; clade C containing the *E. hormaechei* subsp. *oharae* type strain is also Hoffman cluster VI; clade D containing the Hoffmann cluster III type strain (proposed name *E. hormaechei* subsp. *hoffmannii* subsp. nov.) is Hoffmann cluster III; and clade E containing the *E. hormaechei* subsp. *hormaechei* type strain is Hoffmann cluster VII.

While we believe that ANI and other similar measures recently categorized as overall genome related index (OGRI) ^35^ should be used for species/subspecies determination, phenotypic differences due to gene content may play a role particularly for delineation of subspecies. To explore the gene content differences of the *E. cloacae* complex and the *E. hormaechei* subspecies in particular, the pan-genome of the 1,216 *E. cloacae* complex genomes was determined using PanOCT ^36^. The pan-genome generates orthologous gene clusters that delineate which genes are in common between the clades and which genes differentiate the clades ( Supplemental Table 3 and Supplemental Table 4). There were 2,966 genes in “common to all” of the clades (present in 90% of the genomes of each clade). The number of genes “specific to” a clade (present in 90% of the genomes of that clade and in less than 10% of genomes from any other clade) varied from 0 (L) to 465 (V). The number of genes “missing from” a clade (present in less than 10% of the genomes of that clade and present in at least 90% of the genomes of all other clades) varied from 0 (A,C,H,K,O) to 40 (U). The clades which represent named species and subspecies show no qualitative difference in gene content from clades with no named species ( Supplemental Table 4). In particular, clade D which is the proposed *E. hormaechei* subsp. *hoffmannii* has more genes specific to it than 3 of the 4 recognized subspecies. The gene content numbers need to be looked at carefully since they depend on the number of genomes in a clade (T has 187 clade specific genes but this is based on a single genome which means it is really strain specific genes rather than species specific), the distance from other clades (V the most distant clade has 465 specific genes and also has only 3 genomes), and sampling bias such as if most genomes in a clade are from a clonal outbreak. Gene content analysis can also be confounded by misassembly or misannotation of draft genomes which is why we use RefSeq genomes which have passed a quality screen and are consistently annotated. Again we emphasize that ANI as our primary criterium appears to have less of these subjective issues to deal with.

Biochemical and other properties of the *E. hormaechei* subspp. clades have been previously published ^3, 5^ except for clade D. These biochemical properties were used to differentiate between the subspecies but not between other species within the *E. cloacae* complex. With the availability of whole genome sequences and pan-genome analysis tools some of the observed phenotypic traits can be assigned to genetic features, such as the presence or absence of protein coding genes for known metabolic pathways. *E. hormaechei* subsp. *hormaechei* was previously distinguished from *E. hormaechei* subsp. *oharae* and *E. hormaechei* subsp. *steigerwaltii* by growth on dulcitol (a.k.a. galactitol) as the sole carbon source ^3^. This phenotype can be explained by the presence of a *gat* operon ^7, 37^ within all 7 of the *hormaechei* subsp. genomes while none of *oharae*, *steigerwaltii*, or *hoffmannii* genomes have the *gat* operon. In the same genomic location, between the D-galactarate dehydratase gene and the 16S rRNA methyltransferase gene, all of the *steigerwaltii, oharae,* and *hoffmannii* subspp. genomes have a related, but different operon, encoding for N-acetyl galactosamine metabolism (a.k.a., the *aga* operon) ^7, 38^. For *xiangfangensis* most (222 out of 255) of the genomes have the *aga* operon but 33 have the *gat* operon instead. Similarly, *steigerwaltii* isolates can be distinguished from *hormaechei*, *oharae*, *xiangfangensis*, and *hoffmannii* by their ability to grow on adonitol (a.k.a. ribitol) and D(+)-arabitol; both 5 carbon sugar alcohols known as penitols. The *rbt* and *dal* operons known from *Klebsiella aerogenes*, which metabolize ribitol and D(+)-arabitol respectively ^7, 39^, account for this difference where all 325 *steigerwaltii* genomes contain these operons but only 1 hoffmannii and no other hormaechei subspp. genomes do. The gat, aga, and rbt/dal operons are not limited to the E. hormaechei clades but appear in some other *E. cloacae* complex species as shown in Supplemental Table 6. *E. hormaechei* subsp. *hoffmannii* has 25 clade specific genes 10 of which (clusters 28856-28865 Supplemental Table 3) occur as a unit between core clusters (16694-5) and another 6 (15153-15156, 27141-2) occur between core clusters (17653-4). These clusters have no or vague annotation but are intriguing targets to provide functional phenotypic differences.

## Methods

MASH ^32^ is a very fast tool for determining approximate pairwise ANI values given sequenced genomes. A PERL script was used to invoke the following command to generate a set of MASH (version 2.0) sketches of k-mer size 16 for the 1,249 downloaded *Enterobacter* genomes:

*mash sketch -k 16 -o Enter.Sketch.file [List of the Genomes]*

The resulting sketches file was then used to compare all the genomes against each other with an additional PERL script which calls MASH (version 2.0) with the command:

*Mash dist Enter.Sketch.file [List of the Genomes]*

which generated data that could be extracted into an all versus all ANI comparison ( Supplemental Table 5). We used the GGRaSP ^40^ R package (version 1.0) which generated an ultramateric tree by using the R hclust function with average linkage from the distance matrix calculated by subtracting 100 from the MASH ANI results. The result was translated into Newick format with the APE ^41^ R package ( Supplemental File 1) rendered with metadata annotated using the Interactive Tree of Life ^42^ into Figure 1.

Based on the tree 30 genomes were deemed to be outliers and probably not in the *Enterobacter* genus as well as 2 *E. lignolyticus* genomes and 1 *E. timonensis* genome deemed to be outside of the *E. cloacae* complex. These 30 genomes were compared to all genome sequenced bacterial type strains from NCBI RefSeq ( Supplemental Table 2) using MASH which confirmed that these genomes were likely misnamed as *Enterobacter*. The decision to leave *E. lignolyticus* and *E. timonensis* outside of the *E. cloacae* complex was based on two reasons: historically neither has been included in the complex, and there is a quantitative difference in the mean ANI values between genomes of these two species and genomes included in the 22 clades within the complex (last two rows of Table 2). The highest mean ANI for *E. lignolyticus* and *E. timonensis* to genomes included in the 22 clades within the complex is 86.2% for *E. timonensis* to clade S; whereas, the lowest mean ANI within the complex is 86.5% between clades P and U. To further support the decision on what genomes were outliers, we took the 30 outliers, the *E. lignolyticus* and *E. timonensis* type strains, the 23 *E. cloacae* complex type or proxy strains ( Table 1), all type strains from genera with best MASH matches to the 30 outliers ( Supplemental Table 2), and all type strains from other genera closely related to *Enterobacter* and generated pairwise ANI values using PanOCT ( Supplemental Table 7) to build both UPGMA and Neighbor-Joining trees ( Supplemental Figure 2). This analysis supported our decision on what genomes are outliers. One anomaly arose from this analysis: the current type strain genome for *Lelliottia nimipressuralis* currently in GenBank ( **ASM187564v1**) is the same species as the proposed *E. roggenkampii* ( **ASM172980v1**) type strain. The type strain 16S sequence (Z96077) for *Lelliottia nimipressuralis* doesn’t match this purported type strain genome sequence and this genome is an exact duplicate to the previously submitted *Enterobacter* sp. FB ( **ASM80579v1**). The duplicate genomes are from the same submitter and the only reasonable conclusion is that this was a submission error for *Lelliottia nimipressuralis*. This has been reported to NCBI GenBank for resolution ( Supplemental File 2).

From the all versus all MASH ANI comparison GGRaSP was used to generate average linkage clusters and the medoids of those clusters at both the 95% (species) and 98% (subspecies) levels. If type strains existed at the subspecies level those clusters were used ( *E. hormaechei* and *E. cloacae*) otherwise species level clusters were used resulting in 22 clades (A-V). If a type strain genome sequence existed for a clade it was selected otherwise the medoid was selected as a proxy. The one exception for this was clade J where two different type strains existed: *E. asburiae* and *E. muelleri* where both were retained for the typing. These 23 representative genomes were used to “type” all 1,216 *Enterobacter cloacae* complex genomes ( Supplemental Table 1). For typing the best MASH ANI match was used and resolved to either the species or subspecies level. As expected the typing was in complete agreement with the clades in the MASH ANI tree ( Figure 1). The MASH sketches for these 22 clade representatives (after removing the redundant *E. muelleri*) can be used as a fast categorization tool for novel *Enterobacter cloacae* complex genomes.

GGRaSP was similarly used to select the 250 most diverse genomes including the outliers from the 1,249 downloaded genomes while eliminating very closely related genomes. PanOCT ^36, 43^ run at the nucleotide level was used to generate the orthologous clusters for a pan-genome. The primary use of this was to validate the approximate MASH ANI values. PanOCT determines pairwise ANI values by looking at every orthologous cluster shared by a pair of genomes. The percent identity of each match is weighted by the length of the match, summed over all relevant clusters, and divided by the sum of match lengths which is consistent with previous calculations of ANI. Supplemental Figure 1 shows that the MASH ANI estimate is very strongly correlated (98.9) with the PanOCT ANI measurement. For PanOCT ANI values greater than 94% the estimate is very tight (mean error 0.34±0.22) versus less than 94% (1.15±0.70). The clades and tree at the clade level remained the same using PanOCT ANI values.

The reason we use MASH to estimate ANI is that few other tools such as Genome-to-Genome Distance Calculator (GGDC) ^18^ are efficient enough to compute 1249×1249 pairwise comparisons. To our knowledge GGDC is only available as a web based application with a limit of submitting 75 comparisons at one time. MASH is only an approximation of ANI based on sampling but as we showed for species level comparisons (> 94% ANI) provides a quite accurate estimate. For final determination of novel species boundaries MASH should be supported by an exact ANI calculation as we did using PanOCT which determines ANI based on orthologous matches similar to OrthoANI ^44^. Comparison of MASH and PanOCT ANI to GGDC which has been carefully validated with respect to actual laboratory DDH results increases confidence in our methods. We chose four reasonable size datasets to compare GGDC to PanOCT ANI by generating all versus all comparisons omitting self comparisons: 21 of the most diverse of the 1,216 *Enterobacter cloacae* complex genomes as determined by MASH and GGRaSP, 10 *E. hormaechei* genomes chosen similarly, 10 *E. roggenkampii* genomes chosen similarly, and 10 *E. asburiae*/ *E. muelleri* genomes chosen similarly. In order to easily compare GGDC to PanOCT ANI we converted PanOCT ANI into a distance measure d _PANI_ = 1 – (PanOCT ANI/100). GGDC returns three distance measures: Formula 1: length of all HSPs divided by total genome length, Formula 2: sum of all identities found in HSPs divided by overall HSP length, and Formula 3: sum of all identities found in HSPs divided by total genome length. Total genome length is the sum of the two genomes being compared. Formula 1 is a measure of what percentage of the two genomes are shared in common. Formula 2 is basically one variation of how to calculate ANI. Formula 3 is a combination of formulas 1 and 2. The GGDC recommends Formula 2 for draft genomes since it is affected least by genome completeness. The GGDC then uses some statistical modeling to approximate a predicted laboratory DDH value. Supplemental Figure 3 and Supplemental Table 8 shows that for the combined four datasets d _PANI_ is practically indistinguishable from GGDC Formula 2.

For the PanOCT run with 1,216 genomes to determine gene content similarities, PanOCT was run as part of the JCVI pan-genome pipeline in hierarchical fashion with the following batches of genomes run by PanOCT at level 1: (combined 3 *E. mori*, 3 *E. soli*, 8 *E. cancerogenus*, 8 *E. cloacae* complex clade K, 13 *E. cloacae* complex clade L, 11 *E. cloacae* complex clade N, 4 *E. cloacae* complex clade O, 4 *E. cloacae* complex clade P, 5 *E. cloacae* complex clade S, 1 *E. cloacae* complex clade T); (combined 45 *E. cloacae* subsp. *cloacae*, 9 *E. cloacae* subsp. *dissolvens*); (randomly split into 4 groups 169 *E. hormaechei* subsp. *hoffmannii*); (7 *E. hormaechei* subsp. *hormaechei*); (68 *E. hormaechei* subsp. *oharae*); (randomly split into 8 groups 325 *E. hormaechei* subsp. *steigerwaltii*); (randomly split into 6 groups 255 *E. hormaechei* subsp. *xiangfangensis*); (78 *E. asburiae*); (30 *E. bugandensis*); (71 *E. kobei*); (29 *E. ludwigii*); and (70 *E. roggenkampii*). The level 1 clusters were then combined using PanOCT at level 2 and the final output generated using the PanOCT (version 3.27) command line:

*panoct.pl -R matchtable.txt -f genomes.list -g combined.att_file -P combined.fasta -b final_panoct_run -c 0,95*

The diverse 250 genome PanOCT run and the level 1 PanOCT batch runs used the PanOCT (version 3.27) command line:

*panoct.pl -b results -t combined.blast -f genomes.list -g combined.att -P combined.fasta -S yes -L 1 -M Y -H Y -V Y -N Y -F 1.33 -G y -c 0,50,95,100 -T*

The hierarchical PanOCT run of 1,216 genomes produced a matrix of orthologous gene clusters ( Supplemental Table 3) where the rows are clusters and the columns are genomes with the cells containing the RefSeq IDs for the gene from the corresponding genome. This matrix was used to determine genes common to all, specific to, and missing from clades A-V. Individual PanOCT runs were also done for clade J, D, and M. Clade J to insure that PanOCT ANI values confirmed MASH ANI values that *E. asburiae* and *E. muelleri* are the same species which they did and these ANI values were used to determine the 8 subclades at 98% ANI using hierarchical clustering (hclust in R) average linkage. Clade D to confirm the MASH ANI values for *E. hormaechei* subsp. *hoffmannii* which they did. Clade M was done likewise to confirm *E. roggenkampii* which they did.

## Discussion

The Introduction section reviews how the tools for defining a species have evolved. In a recent review of the genus *Mycobacterium*, the authors proposed that any newly defined bacterial species must have a genome sequence and an ANI comparison carried out against existing sequenced type strains to justify a novel species assignment ^45^. ANI analysis should not be relied on in isolation for defining a species since historical or clinical phenotypic distinctions may be important for example in distinguishing between *E. coli* and *Shigela* which by ANI are the same species. However, genome sequencing appears to be outstripping the taxonomic definition of species within some genera. For the 22 clades of the E. cloacae complex identified here 9 do not have named type strains (7 if the two proposed here are adopted). For important pathogens where clinical practice may rely on proper classification the ability to name these clades/species and provide resources for identifying them could be pivotal. Unfortunately, the current established journal for validly publishing bacterial species’ names, IJSEM, insists on phenotypic characterization and deposition of the type strain before naming is valid. This prevents computational based methods from moving quickly. Paradoxically almost all species identifying diagnostic tests are genotype not phenotype based so genotype is good enough for diagnosis but not species definition. Further, delineating what is acceptable to define as a new species is also genotype not phenotype based whether via DDH, marker genes, or more recently ANI. Worse there are no published standards for what defines the minimal set of phenotypic biochemical assays that must be performed. As the *Mycobacterium* review authors state: “The easy and affordable availability of reliable whole-genome sequences raises doubts about the real added value of investigating phenotypic traits when a new species is described. Actually, different taxonomists use their own panels of tests, often not standardized, to produce results of no use for colleagues and absolutely incomprehensible to the community of mycobacteriologists who have dismissed such approach since the ‘90s. For the genus Mycobacterium the major phenotypic traits that cannot be disregarded should include growth rate and pigmentation of colonies, while the classical investigation of biochemical activities is clearly obsolete.”. If there were accepted standards for minimal phenotypic characterization then culture collection repositories could choose to provide the characterization as fee for service or even for free for type strains as an incentive for deposition. With the rapid growth in synthetic genomics capabilities one could argue that the deposition of a high quality complete genome might suffice rather than a culture.

We propose allowing “placeholder” species or subspecies names such as “ *E. cloacae* complex clade S” in order to enable the most robust use of computational and genomic resources for clinical diagnosis. IJSEM currently recognizes provisional species names under the *Candidatus* designation ^46^. *Candidatus* was designed for unculturable organisms where a type strain could not be maintained but phenotypic data is still required to be submitted. This is not a good fit for the case where genome sequences exist and species/subspecies are determined computationally because it was designed for environmental or unculturable samples with limited sequence data but at least some phenotypic or morphological data. We suggest that some similar designation be used for our proposed “placeholder” names. We do not want to computationally assign permanent names with a provisional status, but would rather have the name itself indicate it is provisional and to be replaced when someone does the hard work of depositing a type strain and any required minimal phenotypic information.

In the Results section we noted that the type strains for *E. asburiae* and *E. muelleri* fall within the same clade which could be separated into subspecies by ANI but we declined to do so. For *E. hormaechei* we did propose new subspecies but this was because subspecies for *E. hormaechei* had already been defined. We believe that there must be a cogent reason for delineating beyond the species level. We agree with Chun *et al.* ^35^ who state: “At this stage, we do not have sufficient data to provide a general guideline for defining subspecies using genome data. However, a good practice should involve the following criteria: (i) OGRIs between subspecies and other species should be lower than the species-level cutoff value, (ii) OGRIs between subspecies should be higher than the species-level cutoff, (iii) strains belonging to different subspecies should be genomically coherent and form distinguishable clades by OGRIs and phylogenomic treeing, (iv) subspecies should be differentiated by a sufficient number of phenotypes, and (v) there should be a sound rationale why subspecies should be created and separately recognized, such as showing different host specificity in the case of pathogens.”. An overall genome related index (OGRI) is a computational measure of genome similarity or distance of which ANI is one such. Our ANI analysis possibly fullfill criteria i-iii although given how few strains are in most of the putative subspecies this does not seem robust and criteria iv-v are clearly not met. We only raised the subspecies issue for *E. asburiae* and *E. muelleri* because often in the past when two competing names exist for a species if the type strains can be separated into clear clades they become subspecies. Since the type strains fall into neither of the major clades for this species and certainly do not cleanly divide the species we did not feel this was appropriate.

Computational analysis supports the reassignment of *E. xiangfangensis* to *E. hormaechei* subsp. *xiangfangensis*. We propose to name clade D/Hoffman cluster III as *E. hormaechei* subsp. *hoffmannii* in honor of Harald Hoffmann’s work elucidating the phylogenetic structure of the *E. cloacae* complex ^2^ in particular the subspecies of *E. hormaechei* ^3^. We propose to name clade M/Hoffmann cluster IV *Enterobacter roggenkampii* after Andreas Roggenkamp for his work on elucidating the phylogenetic structure of the *E. cloacae* complex ^2^. The analysis also shows that *E. muelleri* ^33^ is a later heterotypic synonym of *E. asburiae* ^34^ which should take precedence.

### Description of *Enterobacter hormaechei* subsp. *xiangfangensis* subsp. nov., comb. nov.

*E. hormaechei* subsp. *xiangfangensis* (xi.ang.fang.en′sis. N.L. gen. m. adj. *xiangfangensis* pertaining to Xiangfang, a district located in Harbin, Heilongjiang Province, where the bacterium was first isolated).

Basonym: *Enterobacter xiangfangensis* ^5^.

The species description is unchanged from its description as *Enterobacter xiangfangensis* ^5^.

The type strain is strain 10–17 ^T^ (=LMG 27195 ^T^=NCIMB 14836 ^T^=CCUG 62994 ^T^), isolated from traditional sourdough in Heilongjiang Province, China.

The GenBank accessions for the complete genome sequence of *E. hormaechei* subsp. *xiangfangensis* are PRJNA259658, SAMN05581746, ASM172978v1, and CP017183.1.

### Description of *Enterobacter hormaechei* subsp. *hoffmannii* subsp. nov.

*E. hormaechei* subsp. *hoffmannii* (hoff.mannʹi.i. N.L. gen. m. *Hoffmann*, in honor of Harald Hoffmann, a German microbiologist who helped elucidate the phylogenetic structure of the *E. cloacae* complex in particular the subspecies of *E. hormaechei*).

Hoffmann and Roggenkamp ^2^ determined clusters within the *E. cloacae* complex using marker genes, primarily *hsp60*. Hoffman *et al.* ^3^ followed up on three closely grouping clusters to define the three current subspecies of *E. hormaechei* based on DDH and phenotypic tests. Chavda *et al.* ^7^ determined groups for the *E. cloacae* complex using SNPs from whole genome alignments. ANI analysis showed that the Chavda groups were highly similar at levels associated with species or subspecies groupings. This paper performs a more detailed analysis of gene content and ANI across a larger set of genomes supporting the Chavda groups A-E as *E. hormaechei* subspecies. *E. hormaechei* subsp. *hoffmannii* subsp. nov. has similar gene content and ANI characteristics as the previously defined four subspecies.

Hoffmann deposited the type strain, EN-114, for *Enterobacter hormaechei* subsp. *hoffmannii* in Leibniz-Institut DSMZ-Deutsche Sammlung von Mikroorganismen und Zellkulturen GmbH, accession DSM-14563, and recently the strain was also deposited in BCCM/LMG Bacteria Collection, accession LMG-30171. The GenBank accessions for the complete genome sequence are PRJNA259658, SAMN05581748, ASM172974v1, CP017186.1, and CP017187.1.

According to 2, the strain was isolated from the respiratory tract of a clinical patient. The DSMZ database indicates that the sample was isolated prior to 2002 in Bavaria, Germany.

### Description of *Enterobacter roggenkampii* sp. nov.

*E. roggenkampii* (rog.gen.kampʹi.i. N.L. gen. m. *Roggenkamp*, in honor of Andreas Roggenkamp, a German microbiologist who helped elucidate the phylogenetic structure of the *E. cloacae* complex).

Hoffmann and Roggenkamp ^2^ determined clusters within the *E. cloacae* complex using marker genes, primarily *hsp60*. Chavda *et al* ^7^. determined groups for the *E. cloacae* complex using SNPs from whole genome alignments. ANI analysis showed that the Chavda groups were highly similar at levels associated with species or subspecies groupings. *Enterobacter roggenkampii* sp. nov. is the type strain for Hoffmann cluster IV and Chavda group M. This paper performs a more detailed analysis of gene content and ANI across a larger set of genomes supporting the Chavda groups A-R and adding S-V. *E. roggenkampii* sp. nov. has similar gene content and ANI characteristics as previously defined species in the *E. cloacae* complex.

Hoffmann deposited the type strain, EN-117, for *Enterobacter roggenkampii* in Leibniz-Institut DSMZ-Deutsche Sammlung von Mikroorganismen und Zellkulturen GmbH, accession DSM-16690, and recently the strain was also deposited in BCCM/LMG Bacteria Collection, accession LMG-30172. The GenBank accessions for the complete genome sequence are PRJNA259658, SAMN05581750, ASM172980v1, CP017184.1, and CP017185.1.

According to ^2^, the strain was isolated from the stool of a clinical patient. The DSMZ database indicates that the sample was isolated in 2000 in Germany.

The GenBank accessions for the complete genome sequence of *E. hormaechei* subsp. *steigerwaltii* are PRJNA259658, SAMN05581751, ASM172972v1, and CP017179.1.

The GenBank accessions for the complete genome sequence of *E. hormaechei* subsp. *oharae* are PRJNA259658, SAMN05581749, ASM172970v1, and CP017180.1.

## Data availability

The data referenced by this article are under copyright with the following copyright statement: Copyright: © 2018 Sutton GG et al.

Data associated with the article are available under the terms of the Creative Commons Zero "No rights reserved" data waiver (CC0 1.0 Public domain dedication). 

All data underlying the results are available as part of the article and no additional source data are required

## Supplementary material

**Supplemental Table 1. ANI clades compared to MASH best match assignment for 1,216 *Enterobacter cloacae* complex genomes.**

**Supplemental Table 2. MASH typing of 30 outlier genomes falling outside of the *Enterobacter cloacae* complex but labelled as *Enterobacter* in RefSeq.**

**Supplemental Table 3. PanOCT generated orthologous clusters for 1,216 *Enterobacter cloacae* complex genomes. Rows are clusters, columns are genomes, cells contain RefSeq gene identifiers.**

**Supplemental Table 4. Gene counts for genes common to all genomes, specifc to a clade, or missing from a clade.**

**Supplemental Table 5. Pairwise MASH Average Nucleotide Identity (ANI) values for 1,249 genomes labelled *Enterobacter* in RefSeq.**

**Supplemental Table 6. Distribution of *gat*, *aga*, and *rbt/dal* operons across E cloacae complex clades.**

**Supplemental Table 7. PanOCT Average Nucleotide Identity (ANI) pairwise values for 30 outlier genomes and related type species genomes.**

**Supplemental Table 8. Genome distance measure values for representative genomes using GGCD, PanOCT ANI, and MASH.**

**Supplemental Figure 1. Graph of MASH estimated versus PanOCT calculated Average Nucleotide Identity (ANI) for 250 representative genomes.**

**Supplemental Figure 2. Phylogenetic trees of 30 outlier genomes and related type species genomes.**

**Supplemental Figure 3. Graphs of PanOCT ANI, MASH, and GGCD genome distance measures.**

**Supplemental File 1. Newick formatted tree generated from Supplemental Table 5 and used to generate Figure 1.**

**Supplemental File 2. Details about mistaken *Lelliottia nimipressuralis* type strain genome.**
