# Supplementary figures and images for: Enterobacter hormaechei subsp. hoffmannii subsp. nov., Enterobacter hormaechei subsp. xiangfangensis comb. nov., Enterobacter roggenkampii sp. nov., and Enterobacter muelleri is a later heterotypic synonym of Enterobacter asburiae based on computational analysis of sequenced Enterobacter genomes
Source: F1000Res. 2018 Jun 29;7:521. Originally published 2018 May 1. [Version 2] doi: 10.12688/f1000research.14566.2 (PMC6097438; doi:10.12688/f1000research.14566.2)

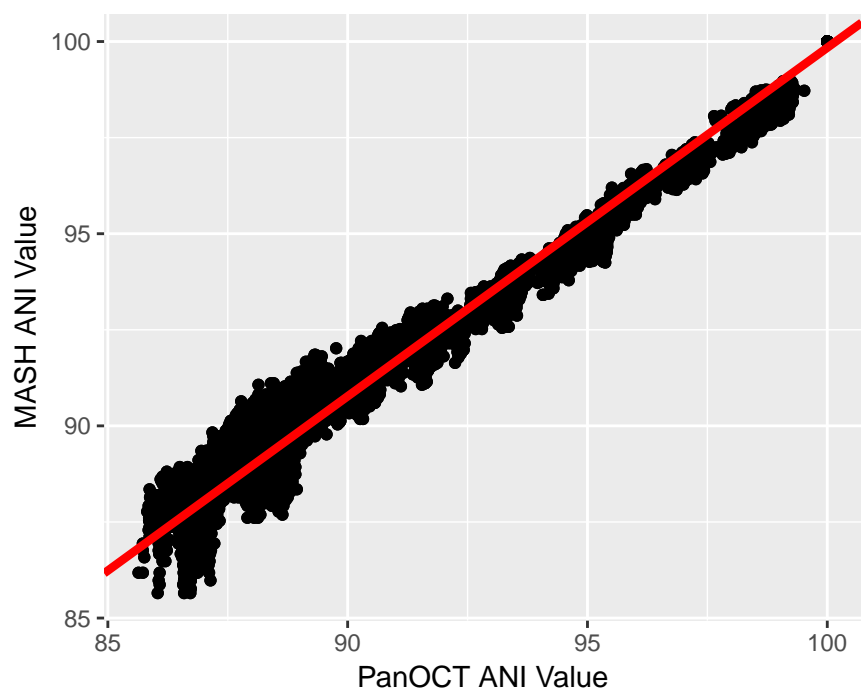

Supplement: Supplementary file 9 [file f1000research-7-16861-s0008.tgz › 70ba1393-18e7-4747-bd71-fe8d3c2936e0.pdf]

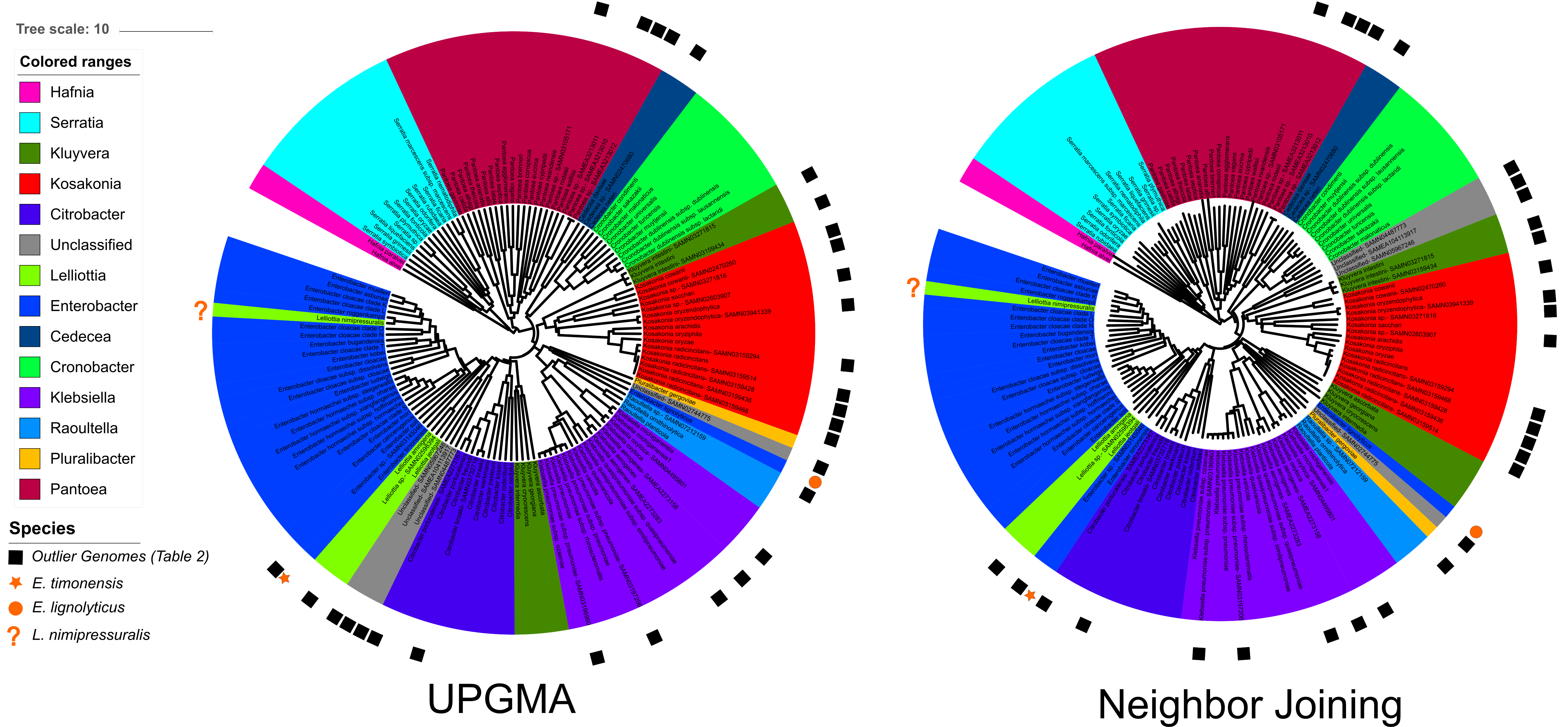

Supplement: Supplementary file 10 [file f1000research-7-16861-s0009.tgz › 3feeca19-c3fd-4396-98ee-45a40cbe26f9.png]

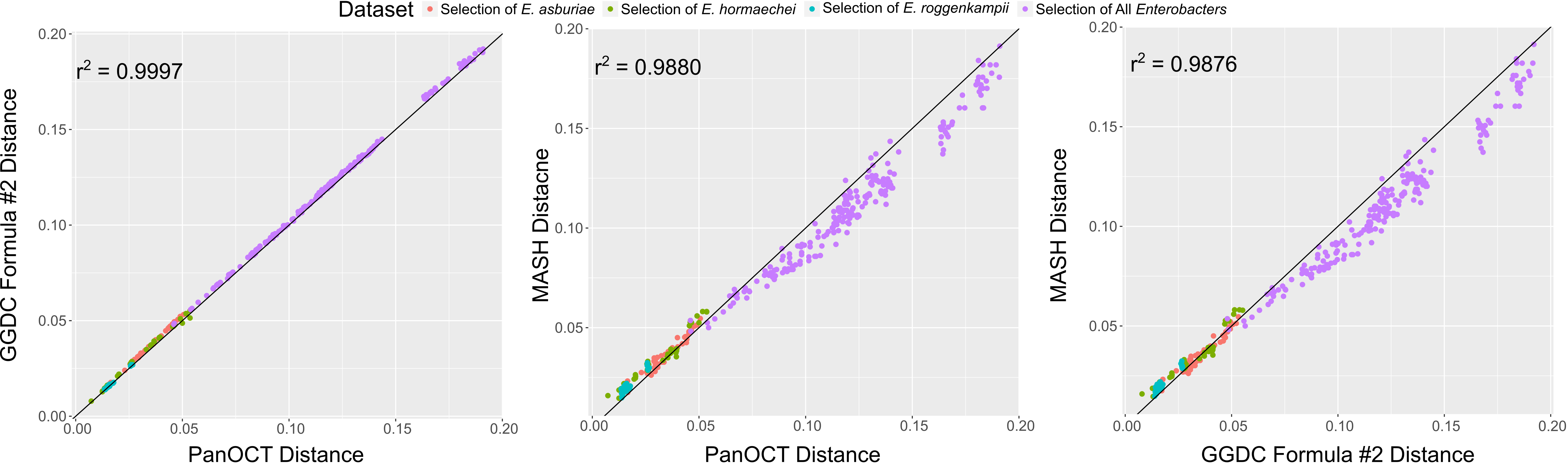

Supplement: Supplementary file 11 [file f1000research-7-16861-s0010.tgz › 31b8aa6a-2e8a-4277-b148-2315e7982e89.png]
