# Supplementary material for: Enterobacter hormaechei subsp. hoffmannii subsp. nov., Enterobacter hormaechei subsp. xiangfangensis comb. nov., Enterobacter roggenkampii sp. nov., and Enterobacter muelleri is a later heterotypic synonym of Enterobacter asburiae based on computational analysis of sequenced Enterobacter genomes
Source: F1000Res. 2018 Jun 29;7:521. Originally published 2018 May 1. [Version 2] doi: 10.12688/f1000research.14566.2 (PMC6097438; doi:10.12688/f1000research.14566.2)
Supplement: Supplementary file 13 [file f1000research-7-16861-s0012.tgz › 10cc6beb-3c65-47a6-959e-8fa263f819df.docx]

**Supplementary File 2**

The type strain genome for *Lelliottia nimipressuralis* currently in GenBank (**ASM187564v1**) is the same species as the proposed *E. roggenkampii* (**ASM172980v1**). The type strain 16S sequence (Z96077) for *Lelliottia nimipressuralis* doesn’t match this genome sequence and this genome is an exact duplicate with the same number of contigs, same N50, same genome size and 100% identity match to the previously submitted *Enterobacter* sp. FB (**ASM80579v1**). The duplicate genomes are from the same submitter and the only reasonable conclusion is that this was a submission error for *Lelliottia nimipressuralis*. This has been reported to NCBI GenBank for resolution. Details below:

LOCUS Z96077 1498 bp DNA linear BCT 06-JUN-2003

DEFINITION Enterobacter nimipressuralis LMG 10245-T 16S ribosomal RNA.

ACCESSION Z96077

VERSION Z96077.1

KEYWORDS 16S ribosomal RNA.

SOURCE Lelliottia nimipressuralis

ORGANISM [Lelliottia nimipressuralis](https://www.ncbi.nlm.nih.gov/Taxonomy/Browser/wwwtax.cgi?id=69220)

Bacteria; Proteobacteria; Gammaproteobacteria; Enterobacterales;

Enterobacteriaceae; Lelliottia.

REFERENCE 1

AUTHORS Hauben,L., Moore,E.R., Vauterin,L., Steenackers,M., Mergaert,J.,

Verdonck,L. and Swings,J.

TITLE Phylogenetic position of phytopathogens within the

Enterobacteriaceae

JOURNAL Syst. Appl. Microbiol. 21 (3), 384-397 (1998)

**ASM187564v1**

**Organism name:**

[Lelliottia nimipressuralis (enterobacteria)](https://www.ncbi.nlm.nih.gov/Taxonomy/Browser/wwwtax.cgi?mode=Info&id=69220&lvl=3&lin=f&keep=1&srchmode=1&unlock)

**Infraspecific name:**

Strain: CIP 104980

**BioSample:**

[SAMN05787348](https://www.ncbi.nlm.nih.gov/biosample/SAMN05787348/)

**Submitter:**

Zhejiang Sci-Tech University

**Date:**

2016/11/10

**Assembly level:**

Contig

**Genome representation:**

full

**Relation to type material:**

assembly from type material

**GenBank assembly accession:**

GCA_001875645.1 (latest)

**RefSeq assembly accession:**

GCF_001875645.1 (latest)

**RefSeq assembly and GenBank assembly identical:**

yes

**WGS Project:**

[MKER01](https://www.ncbi.nlm.nih.gov/nuccore/MKER00000000.1/)

**Assembly method:**

HGAP v. 3.0

**Expected final version:**

yes

**Reference guided assembly:**

de-novo

**Genome coverage:**

78.0x

**Sequencing technology:**

PacBio

And

**ASM80579v1**

**Organism name:**

[Enterobacter sp. FB (enterobacteria)](https://www.ncbi.nlm.nih.gov/Taxonomy/Browser/wwwtax.cgi?mode=Info&id=1571816&lvl=3&lin=f&keep=1&srchmode=1&unlock)

**Infraspecific name:**

Strain: FB

**BioSample:**

[SAMN03174119](https://www.ncbi.nlm.nih.gov/biosample/SAMN03174119/)

**Submitter:**

Chengdu Military General Hospital

**Date:**

2014/12/19

**Assembly level:**

Contig

**Genome representation:**

full

**GenBank assembly accession:**

GCA_000805795.1 (latest)

**RefSeq assembly accession:**

GCF_000805795.1 (latest)

**RefSeq assembly and GenBank assembly identical:**

yes

**WGS Project:**

[JSZC01](https://www.ncbi.nlm.nih.gov/nuccore/JSZC00000000.1/)

**Assembly method:**

HGAP protocol,SMRT Analysis v. 2.2.0

**Expected final version:**

yes

**Reference guided assembly:**

de-novo

**Genome coverage:**

78.14x

**Sequencing technology:**

PacBio
