# Supplementary material for: Enterobacter hormaechei subsp. hoffmannii subsp. nov., Enterobacter hormaechei subsp. xiangfangensis comb. nov., Enterobacter roggenkampii sp. nov., and Enterobacter muelleri is a later heterotypic synonym of Enterobacter asburiae based on computational analysis of sequenced Enterobacter genomes
Source: F1000Res. 2018 Jun 29;7:521. Originally published 2018 May 1. [Version 2] doi: 10.12688/f1000research.14566.2 (PMC6097438; doi:10.12688/f1000research.14566.2)
Supplement: Peer review history

# Peer review: Referee response for version 2

**DOI:** 10.5256/f1000research.16861.r35633

**Published:** 2018-07-13

**Authors:** Trinad Chakraborty, Swapnil Doijad

**Affiliations:** 1 Institute of Medical Microbiology, Justus-Liebig University Giessen, Giessen, Germany

From our point of view, digital DDH (dDDH) calculated by GGDC tool correlates better with empirical DDH than the various implementations of ANI (Meier-Kolthoff 2014 ^1^, Meier-Kolthoff et al., 2013 ^2^) and thus dDDH can safely be preferred over ANI. GGDC estimates at 70% dDDH are widely accepted, have repeatedly proven to be accurate, and is recommended for the discrimination of the species by bacterial taxonomical experts (and not the preliminary ANI derived from GGDC algorithm) (Chun et al., 2018 ^3^).

 In bacterial taxonomy, only type strains are valid, while strains belong to phylogenomically diverse clades are less important during defining a species. As there are limitations in the use of the GGDC tool to compare a large number of strains, it would have been more logical to estimate GGDC-dDDH values (only) for the type strains for which the ANI cutoff is ambiguous.

 The GGDC tool shows *E. xiangfangensi*s and *E. hormaechei* are in fact two different species (DDH estimate (GLM-based): 59.80% [56.9 - 62.5%]). Indeed, there is only a 12% probability that these two type strain can be classified as subspecies of a either species. Extension of this estimate to clade level indicates clade A-D and E are two different species and represents *E. xiangfangensi*s and *E. hormaechei, *respectively. Just because several strains of clade A-D were historically identified as *E. hormaechei *subspecies, there is no reason to ignore the recently validated *E. xiangfangensis *species.

 Currently, phenotypic identification remains the gold standard for identification of microorganisms in standard diagnostic laboratories and provides the bulk of the data for taxonomic classification. WGS is presently largely done in research laboratories or as pilot endeavors in specialized diagnostic laboratories. As genotype-phenotype correlations are at present incomplete, current classification schemes would give phenotypic data priority.

We have read this submission. We believe that we have an appropriate level of expertise to confirm that it is of an acceptable scientific standard.

## References

[1] Meier-KolthoffJPKlenkHPGökerM: Taxonomic use of DNA G+C content and DNA-DNA hybridization in the genomic age. *Int J Syst Evol Microbiol*.2014;64(Pt 2) : 10.1099/ijs.0.056994-0 352-6 10.1099/ijs.0.056994-0 24505073

[2] Meier-KolthoffJPAuchAFKlenkHPGökerM: Genome sequence-based species delimitation with confidence intervals and improved distance functions. *BMC Bioinformatics*.2013;14: 10.1186/1471-2105-14-60 60 10.1186/1471-2105-14-60 23432962PMC3665452

[3] ChunJOrenAVentosaAChristensenHArahalDRda CostaMSRooneyAPYiHXuXWDe MeyerSTrujilloME: Proposed minimal standards for the use of genome data for the taxonomy of prokaryotes. *Int J Syst Evol Microbiol*.2018;68(1) : 10.1099/ijsem.0.002516 461-466 10.1099/ijsem.0.002516 29292687

# Response

**Published:** 2018-07-17

**Authors:** Granger Sutton

**Affiliations:** J. Craig Venter Institute, USA

We agree that dDDH (Refs 1,2) has been shown to correlate slightly better with laboratory DDH than ANI does. We do not agree that this should provide the basis for preferring dDDH over ANI as the basis for determining species or subspecies level relatedness. The proposed criteria from Chun et al (Ref 3) also do not give a preference for dDDH over ANI as an OGRI. The question we need to ask is what defines a species? Genome relatedness is certainly a primary component of that and because laboratory DDH was the first method for calculating genome relatedness it became the gold standard but with current  genome sequencing there is no reason for it to remain the gold standard. dDDH combines shared genomic content with the ANI of the shared genomic content. PanOCT computes multiple pairwise relatedness measures: two of which are the ANI of orthologous genes and the Jaccard similarity of the gene content. We have shown (Ref 4) that the gene content similarity measure can be significantly affected by horizontally transferred genes such as plasmids which raises the question of whether that should be part of a species relatedness measure. Chun et al (Ref 3) argue that at levels above species and certainly above genera that OGRI measures are not useful and rather that a set of core genes with low horizontal transfer potential be used for phylogenetic tree construction. This is much more consistent with ANI which tends to measure the core gene similarity rather than dDDH which includes variable gene content. We believe that evolutionary relatedness including species definitions is best measured with ANI while gene content provides a somewhat orthogonal measurement to capture horizontal transfer events. We recognize that horizontal transfers are also evolutionary events and strongly correlated with ANI hence the "somewhat orthogonal". We welcome the discussion of what should define a species and understand that the views of Drs. Chakraborty and Doijad are as valid as our own.

 Using DDH Hoffman et al (Ref 5) showed that *Enterobacter hormaechei* subsp. *oharae*, *E. hormaechei *subsp. *hormaechei*, and *E. hormaechei* subsp. *steigerwaltii* are the same species: "The close DNA-DNA relatedness within clusters VI and VII was reflected by ΔTm values below 0.5. The relatively higher heterogeneity of cluster VIII was indicated by higher within-group ΔTm values of up to 2.7. By evaluating the DNA relatedness among the clusters, we found that clusters VI and VIII are closely related (mean ΔTm value = 2.2), while a relatively longer distance for E. hormaechei cluster VII from the members of clusters VI and VIII was indicated by the mean ΔTm value of 4.0. However, all three genetic clusters could still be assigned to the same species (14). They could be genetically distinguished from the other species of the E. cloacae complex, which had ΔTm values of 5.6 to 10.3 (Table 2).". Unfortunately they did not report DNA-DNA relatedness values but only ΔTm values. They did cite previous work which gave DNA-DNA relatedness: "Davin-Regli et al. ( 4) reported an outbreak with an “ *E. cloacae* strain with the *E. hormaechei* genotype” but an aberrant biotype. The strain exhibited all of the characteristics of *E. hormaechei* and was 80% related to the type strain in DNA-DNA reassociation experiments but was positive for growth on D-sorbitol and α-D-melibiose. Obviously, this outbreak was caused by a strain of genetic cluster VI. Hence, these studies are in agreement with our observation that genetic clusters VI and VIII belong to the species *E. hormaechei* ( 4, 6).". We agree that by ANI and dDDH that *E. hormaechei* subsp. *hormaechei* is borderline at best to be grouped as the same species as the other *E. hormaechei* subspecies but Drs. Chakraborty and Doijad cannot have it both ways. Hoffman et al showed phenotypic data supporting there grouping of the subspecies and delineation from other subspecies as well as genotypic support using marker genes which has since been used in clinical papers to differentiate the subspecies from each other and other species. Certainly one could propose making these separate species but the bar for undoing historical precedent is much higher than arguing that the ANI or dDDH values are borderline.

 Drs. Chakraborty and Doijad state: "Currently, phenotypic identification remains the gold standard for identification of microorganisms in standard diagnostic laboratories and provides the bulk of the data for taxonomic classification. WGS is presently largely done in research laboratories or as pilot endeavors in specialized diagnostic laboratories. As genotype-phenotype correlations are at present incomplete, current classification schemes would give phenotypic data priority.". Not being clinicians we are not sure if this is true but based on our reading of the literature if it is true it is likely to not be true in the near future. We are not against phenotypic characterization if it is economical and reliable. We look forward to a robust discussion of the pros and cons of phenotyic versus genotypic diagnostic methods. Regardless, assignment of species and species delineation has long been genotype based since DDH is a genotypic measure as well as marker genes and OGRI. We are not against phenotypic characterization of type strains although one could argue that this only really makes sense if a clade of strains of the same species is characterized to evaluate variability. We reached out to DSMZ to inquire about phenotypic characterization services which they are willing to provide at some level on a case by case basis but they could not tell us what minimal characterization is necessary for a type strain. Perhaps Drs. Chakraborty and Doijad could intervene on our behalf with DSMZ and have the appropriate characterization performed and placed in the DSMZ supported  “The Bacterial Diversity Metadatabase” (BacDive). This could be the first step towards some form of phenotypic characterization standard for type strains.

 1. Meier-Kolthoff JP, Klenk HP, Göker M: Taxonomic use of DNA G+C content and DNA-DNA hybridization in the genomic age. *Int J Syst Evol Microbiol*. 2014; **64** (Pt 2): 352-6 PubMed Abstract | Publisher Full Text 

 2. Meier-Kolthoff JP, Auch AF, Klenk HP, Göker M: Genome sequence-based species delimitation with confidence intervals and improved distance functions. *BMC Bioinformatics*. 2013; **14**: 60 PubMed Abstract | Publisher Full Text 

 3. Chun J, Oren A, Ventosa A, Christensen H, Arahal DR, da Costa MS, Rooney AP, Yi H, Xu XW, De Meyer S, Trujillo ME: Proposed minimal standards for the use of genome data for the taxonomy of prokaryotes. *Int J Syst Evol Microbiol*. 2018; **68** (1): 461-466 PubMed Abstract | Publisher Full Text 

 4. Chavda KD, Chen L, Fouts DE, *et al.*: Comprehensive Genome Analysis of Carbapenemase-Producing *Enterobacter* spp.: New Insights into Phylogeny, Population Structure, and Resistance Mechanisms. *mBio.*2016; **7**(6): pii: e02093-16. PubMed Abstract | Publisher Full Text | Free Full Text

 5. Hoffmann H, Stindl S, Ludwig W, *et al.*: *Enterobacter hormaechei* subsp. *oharae* subsp. nov., *E. hormaechei*subsp. *hormaechei* comb. nov., and *E. hormaechei* subsp. *steigerwaltii* subsp. nov., three new subspecies of clinical importance. *J Clin Microbiol.* 2005; **43**(7): 3297–303. PubMed Abstract | Publisher Full Text | Free Full Text

# Peer review: Referee response for version 2

**DOI:** 10.5256/f1000research.16861.r35632

**Published:** 2018-07-12

**Authors:** Mark J. Pallen

**Affiliations:** 1 Quadram Institute, Norwich, UK

I am happy with the changes.

I have read this submission. I believe that I have an appropriate level of expertise to confirm that it is of an acceptable scientific standard.

# Peer review: Referee response for version 1

**DOI:** 10.5256/f1000research.15853.r34159

**Published:** 2018-06-07

**Authors:** Trinad Chakraborty, Swapnil Doijad

**Affiliations:** 1 Institute of Medical Microbiology, Justus-Liebig University Giessen, Giessen, Germany

Considerable genome data is now available for isolates of many members of the family *Enterobacteriaceae*. As we move away from well-defined species such as *E. coli *and *Salmonella, *taxonomic assignments become blurred and there is now a great need to develop standardized tools for proper classification. A particular case is that of the species *Enterobacter, *where only 12 of the 35 historically classified species in this genus are valid.

 The present manuscript reevaluates taxonomic allocation of members of the *Enterobacter cloacae* complex using whole genome sequences (WGS). It is important to remember that the dataset comprises primarily of draft genome sequences of varying quality and with only a very small number representing truly closed genomes.

 Isolates of the *E. hormaechei *complex are often associated with clinical disease. Based on the data from this study there are now two novel subspecies of *E. hormaechei* designated as *E. hormaechei* subsp. *hoffmannii* and *E. hormaechei *subsp. *xiangfangensis *respectively. In addition, a new species *E.* *roggenkampii *is proposed. Overall the study predicts the existence of 7 additional species within the genus *Enterobacter*.

 The bulk of the analysis is based on a single tool viz. MASH-based ANI and is supplemented by the panOCT tool developed by the authors. The authors should consider the use of additional software tools to determine the overall genome-related index (OGRI).

 Specific comments:

- Clade A-E represent the five subspecies of *E. hormaechei. *The average nucleotide identity (ANI) for the clades A-D and E are at the borderline ANI-species definition.
- In view of the fact that data is based mainly on draft genomes, the utility of supportive assignments based on the total numbers of unique genes must be considered carefully.
- For such closely related clades, multi-tool-based analysis of taxonomy are helpful to reassure the claims. To support the species/subspecies distinction, particularly for those closely related clades, the use of widely used taxonomic tools such as the digital DNA-DNA hybridization tool, GGDC should be employed to strengthen the claims. 
- ANI values can vary when using different calculation tools as for e.g. with JSpecies and ANI calculator. The use of MASH algorithm leads to minor variation in ANI values and makes the borderline species definitions presented here difficult to interpret. 
- To confirm separation of *E. timonensis *and *E. lignolyticus *from the genus *Enterobacter*, comparison with members of the closest genera (for e.g., *Klebsiella, Citrobacter *etc.) should be added.

Finally, biochemical and fermentation characteristics are key indicators for phenotypic characterization of isolates in diagnostic laboratories.

 The final paragraph on biochemical properties is inadequate and could lead to confusion of phenotypes and undo the very purpose of the proposed classification scheme. Thus the *gat *operon is not exclusive to *E. hormaechei *subspecies *hormaechei* as stated, but is also present for e.g. in type strain *E. bugandensis *EB-247 ^T^ *.*

We have read this submission. We believe that we have an appropriate level of expertise to confirm that it is of an acceptable scientific standard, however we have significant reservations, as outlined above.

# Response

**Published:** 2018-06-26

**Authors:** Granger Sutton

**Affiliations:** J. Craig Venter Institute, USA

We thank Dr. Pallen for the thoughtful review and respond to issues below.

 “I don't see the need for the separate Introduction and Background sections. According to the guidelines for authors, papers in this journal should follow the usual IMRAD format, so I think that the two sections should simply become sub-sections of the Introduction, perhaps with brief explanatory headers.”

 We removed the Background and Conclusion section headings to conform to the IMRAD format.

 “I am not sure why the authors abdicate responsibility for determining whether "8 subclades of E. asburiae should be treated as subspecies". Why not roll their approach out to cover these lineages too?”

 We now address this in the Discussion section.

 “The authors discuss the concept of "placeholder" species and subspecies in the Discussion, but fail to mention the "Candidatus" designation, which is recognised by the current bacterial taxonomy apparatchiks:

 http://ijs.microbiologyresearch.org/content/journal/ijsem/10.1099/00207713-45-1-186

 https://en.wikipedia.org/wiki/Candidatus

 They should include some discussion of this designation that includes a recognition of its major shortcoming in requiring phenotypic data in addition to genome sequence.”

 We thank Dr. Pallen for pointing this out to us and have included this in the Discussion section.

 We thank Dr. Chakraborty for the thoughtful review and respond to issues below.

 “The bulk of the analysis is based on a single tool viz. MASH-based ANI and is supplemented by the panOCT tool developed by the authors. The authors should consider the use of additional software tools to determine the overall genome-related index (OGRI).” and

 “For such closely related clades, multi-tool-based analysis of taxonomy are helpful to reassure the claims. To support the species/subspecies distinction, particularly for those closely related clades, the use of widely used taxonomic tools such as the digital DNA-DNA hybridization tool, GGDC should be employed to strengthen the claims.”

 We have included the comparison of GGDC to MASH and PanOCT ANI in the Methods section.

 “Clade A-E represent the five subspecies of *E. hormaechei*. The average nucleotide identity (ANI) for the clades A-D and E are at the borderline ANI-species definition.”

 This is certainly true but is also true of the already existing E. hormaechei subspecies: clade B *E. hormaechei* ssp. *steigerwaltii*, clade C *E. hormaechei* ssp. *oharae*, and clade E *E. hormaechei ssp. hormaechei*. While in the absence of previous taxonomic assignments one might choose to be reluctant to combine clades B, C, and E into a single species based on ANI because they have already been grouped as a species the borderline ANI values are not strong enough to argue for changing this. Given this adding clades A and D to *E. hormaechei* is strongly confirmed by the ANI values between clades A, B, C, and D.

 “In view of the fact that data is based mainly on draft genomes, the utility of supportive assignments based on the total numbers of unique genes must be considered carefully.”

 We have noted this concern in the results section. Gene content is not a primary consideration in our proposed new species designation but rather a possible reason to delineate at the subspecies level. In our experience most recent draft genome sequences are of high quality and the RefSeq genomes we used are screened by NCBI to meet certain quality requirements. Draft genome breaks tend to be at and due to repetitive elements such as transposons which would not affect the representation of most genes. We also try to take this into account by using a 90% rather than a 100% threshold.

 “ANI values can vary when using different calculation tools as for e.g. with JSpecies and ANI calculator. The use of MASH algorithm leads to minor variation in ANI values and makes the borderline species definitions presented here difficult to interpret.”

 ANI values for the newly proposed type strains were backed up by PanOCT ANI and now by GGDC and are not borderline except as consistent with previous taxonomy.

 “To confirm separation of *E. timonensis* and *E. lignolyticus* from the genus Enterobacter, comparison with members of the closest genera (for e.g., *Klebsiella*, *Citrobacter* etc.) should be added.”

 We have added this analysis to the Methods section.

 “Finally, biochemical and fermentation characteristics are key indicators for phenotypic characterization of isolates in diagnostic laboratories.”

 As the paper mentions we are not opposed to the biochemical characterization of type strains but need a standard that can be implemented by culture collections so that computationalists can acquire this data. The DSMZ for instance supports doing some of this characterization but does not claim it to be standard. In addition, DSMZ supports storing this characterization data in “The Bacterial Diversity Metadatabase” (BacDive) such as for the *E. bugandensis* type strain ( https://bacdive.dsmz.de/strain/132404). What is interesting is that most biochemical characterization is not used to define a species in current practice. Researchers no longer collect phenotypic features and cluster based on a feature vector. Rather, genotypic characteristics are captured such as 16S or hsp60 or rpoB or WGS which are used to define a cluster of strains and then phenotypic characterization of those strains is performed and used as part of the species definition no matter how divergent those features may be. Computational taxonomy provides a structure by which strains can be clustered, named, referenced, discussed and compared to related clades. Biologists should follow up on clinically or otherwise interesting clades. We are not sure whether Dr. Chakraborty is arguing for historical consistency in what characterization is minimally required for a type strain or is arguing that there is little or no value in computational taxonomy without phenotypic characterization because it is required for clinical diagnosis. We would disagree with both since with the advent of whole genome sequences  (or even DDH) phenotype is not needed to define species and clinical diagnosis can be done with molecular markers.

 “The final paragraph on biochemical properties is inadequate and could lead to confusion of phenotypes and undo the very purpose of the proposed classification scheme. Thus the gat operon is not exclusive to *E. hormaechei* subspecies *hormaechei* as stated, but is also present for e.g. in type strain *E. bugandensis* EB-247T.”

 We apologize for being unclear. We were summarizing what is already in the literature for distinguishing *E. hormaechei* subspecies from each other. We have been more precise and clarified this issue in the Results section.

# Peer review: Referee response for version 1

**DOI:** 10.5256/f1000research.15853.r33699

**Published:** 2018-05-17

**Authors:** Mark J. Pallen

**Affiliations:** 1 Quadram Institute, Norwich, UK

This is in general a well written and well argued paper that represents a valuable addition to attempts to bring bacterial taxonomy into the genomic age. I can find no fault with the methodologies used nor with the general interpretation of results. I agree with the authors that all future bacterial taxonomy and nomenclature should be based on genomic data and they have fallen in line with an emerging consensus of how to make that work using ANI.

 It is clear that bacterial taxonomy is broken and needs fixing and the only suitable response to the tyranny of The International Committee on Systematic Bacteriology is subversion by publishing papers like this that ignore its ridiculous and outdated requirements.

 To quote Darwin: "Our classifications will come to be, as far as they can be so made, genealogies"

 I have just a handful of minor criticisms/suggestions for improvement:

1. I don't see the need for the separate Introduction and Background sections. According to the guidelines for authors, papers in this journal should follow the usual IMRAD format, so I think that the two sections should simply become sub-sections of the Introduction, perhaps with brief explanatory headers.
2. I am not sure why the authors abdicate responsibility for determining whether "8 subclades of E. asburiae should be treated as subspecies". Why not roll their approach out to cover these lineages too?
3. The authors discuss the concept of "placeholder" species and subspecies in the Discussion, but fail to mention the "Candidatus" designation, which is recognised by the current bacterial taxonomy apparatchiks:
http://ijs.microbiologyresearch.org/content/journal/ijsem/10.1099/00207713-45-1-186
https://en.wikipedia.org/wiki/Candidatus
  They should include some discussion of this designation that includes a recognition of its major shortcoming in requiring phenotypic data in addition to genome sequence.

I have read this submission. I believe that I have an appropriate level of expertise to confirm that it is of an acceptable scientific standard.

# Response

**Published:** 2018-05-17

**Authors:** Granger Sutton

**Affiliations:** J. Craig Venter Institute, USA

The three reviewer suggestions all have merit and we will try to address them once more reviews are received. 1) We can certainly conform to IMRaD by using subheadings. 2) The issue of species versus subspecies should be addressed in the discussion. Our feeling was that when it is already problematic to validly publish names for species it is even more burdensome to do so for subspecies. What is the appropriate criteria to go to the trouble to differentiate subspecies: clinical significance, number of exemplars of each subspecies, and/or amount of core gene content difference between subspecies (this can only be determined once there are enough exemplars of each subspecies)? 3) We were unaware of the Candidatus designation and appreciate this being pointed out. While it does not appear to be a good fit for the case where genome sequences exist and species/subspecies are determined computationally since it was designed for environmental or unculturable samples with limited sequence data but at least some phenotypic or morphological data, it does suggest that some similar designation be used for "placeholder" names. We do not want to assign potentially permanent names with a notation indicating they are provisional but would like the name itself to indicate it is provisional and to be replaced when someone does the hard work of depositing a type strain and any required minimal phenotypic information. Again we should address this in the discussion.
